# Monoallelic Germline *TSC1* Mutations Are Permissive for T Lymphocyte Development and Homeostasis in Tuberous Sclerosis Complex Individuals

**DOI:** 10.1371/journal.pone.0091952

**Published:** 2014-03-14

**Authors:** Karolina Pilipow, Veronica Basso, Nicola Migone, Anna Mondino

**Affiliations:** 1 Lymphocyte Activation Unit, Immunology, Transplantation and Infectious Disease Division, San Raffaele Scientific Institute, Milano, Italy; 2 Università Vita-Salute San Raffaele, Milano, Italy; 3 Department of Genetics, Biology and Biochemistry, University of Torino, and Medical Genetics, Azienda Ospedaliero-Universitaria San Giovanni Battista, Torino, Italy; Johns Hopkins University School of Medicine, United States of America

## Abstract

Germline and somatic biallelic mutations of the Tuberous sclerosis complex (*TSC*) *1* and *TSC2* gene products cause TSC, an autosomal dominant multifocal hamartomatosis with variable neurological manifestations. The consequences of TSC1 or TSC2 loss in cells of hematopoietic origin have recently started to be unveiled in mice and showed to hinder the development of proper T cell immunity. To date, the consequences of germline *TSC1* mutations and/or its loss in mature human T cells remain to be determined. To address these issues, we analyzed subset representation, phenotype and responsiveness to mitogens in T cells from patients with inherited monoallelic *TSC1* mutations, and induced shRNA-mediated TSC1 down-regulation in primary and transformed human T cells. We report that, the distribution of peripheral CD4 and CD8 T cell subsets, their cytokine-secretion profile, and responsiveness to *in vitro* stimulation were largely preserved in TSC subjects with monoallelic *TSC1* germline mutations when compared to healthy controls. Sufficient levels of hamartin and tuberin and proper control of mTOR-dependent signaling in primary T cells from TSC subjects best explained this. In contrast, shRNA-induced down-regulation of *TSC1*, likely mimicking biallelic inactivation of *TSC1*, compromised hamartin and tuberin expression and mTORC2/AKT/FoxO1/3 signaling causing both primary and transformed T cells to die by apoptosis. Thus, our results indicate that, while one functional *TSC1* allele preserves human T lymphocytes development and homeostasis, TSC1 acute down-regulation is detrimental to the survival of both primary and transformed T cells.

## Introduction

The Tuberosis Sclerosis Complex (TSC) is a heterodimer formed by TSC1, also known as hamartin, and TSC2, also known as tuberin, lying at the crossroad of multiple signaling pathways [1]. The TSC complex regulates the mammalian Target Of Rapamycin (mTOR) complex 1 (mTORC1)- and mTORC2-dependent signaling and coordinates inputs from growth factors and energy availability, critical for the regulation of cell quiescence, proliferation and survival. Mutations in either *TSC1* (on chromosome 9q34) or *TSC2* (on chromosome 16p13.3) cause an autosomal dominant disease, TSC, with high penetrance and variability [2], which affects one in 10.000 individuals in the general population, and one in 6.800 in the pediatric age group [2], [3], [4]. One-third of TSC cases are inherited, while two-thirds of all cases are caused by *de novo* mutations. Mutations in the *TSC1/TSC2* genes generally cause characteristic brain lesions called tubers, and widespread benign, focal malformations called hamartomas, which comprise nonmalignant cells exhibiting abnormal proliferation and differentiation, which are found in a variety of organs and tissues, including skin and kidney [5]. Common lesions include renal angiomyolipomas, renal cysts, cardiac rhabdomyomas, facial angiofibromas, periungual fibromas, retinal hamartomas, and pulmonary lymphangioleiomyomas [6], [7]. As a consequences of tuber formation within the cerebral cortex [8], TSC subjects present variable neurological symptoms including infantile spasms, intractable epilepsy and cognitive disabilities [6], [7]. Loss of heterozygosity (LOH) has been formally demonstrated in hamartomas in the skin, kidney, liver, lung, and heart, and reflects a “2-hit” mutational mechanism due to the combined effect of germline and somatic mutations [9], [10], [11]. Whether LOH does occur in tubers has been debated [9], [12], [13], [14]. Biallelic *TSC* gene inactivation was indeed found in giant cells, but proved to be the result of distinct germline and somatic mutational events [15]. Biallelic *TSC* gene inactivation results in elevated mTORC1 signaling and attenuated mTORC2 signaling [10], [13], [14], [16], [17]. In addition to gene inactivation, alternative mechanisms, such as differences in allele specific mRNA expression or haploinsufficiency have also been suggested to influence neuronal structure and function [18], [19].

To date, whether neurological manifestation of TSC exerts non cell-autonomous effects on the development of immune competence or whether *TSC1/2* germline mutations have cell autonomous effects on T cell maturation and/or function remains to be determined. We started addressing this issue, given the notion that conditional biallelic inactivation of *Tsc1* in hematopoietic cell precursors [20] and in developing thymocytes [21], [22], [23], [24] hindered cell quiescence and survival. To this aim, we characterized T cell subsets representation and function in individuals with defined monoallelic germline *TSC1* mutations. We also analyzed the effect of shRNA-mediated inactivation of TSC1 in primary and transformed human T cells, and compared results with those obtained with mouse T cells with mono and biallelic *Tsc1* inactivation. We report that, while one functional *TSC1* allele in TSC subjects is sufficient to preserve normal T cell representation, function, and adaptive recall responses, TSC1 down-regulation leads to deregulated mTOR signaling and apoptotic cell death.

## Results

### TSC individuals with inherited *TSC1* mutations reveal normal representation of mature T cell subsets

We analyzed peripheral blood mononuclear cells (PBMC) from individuals of two independent TSC families with defined monoallelic germline *TSC1* mutations. The first family (Pt 1-2) was characterized by a previously unrecognized Pro substitution at invariant Leu residue 129 (L129P). While this residue is evolutionary conserved among species and found non-mutated in 6503 exome sequences (Exome variant server; http://evs.gs.washington.edu/EVS/), the Leu to Pro mutation was directly linked to the onset of hamartomas in kidney, lung and submandibular region, with a cumulative logarithm of the odds (LOD) score >3 of a large number of tested patients belonging to the same family (Migone et al., manuscript in preparation). According to four different prediction softwares (Pmut; http://mmb.pcb.ub.es/PMut; MUpro: http://www.ics.uci.edu/~baldig/mutation [25]; SIFT; http://sift.jcvi.org and PolyPhen-2: http://genetics.bwh.harvard.edu/pph2) the non-conservative Leu129Pro substitution allowed for protein expression (Figure S1), but is predicted to damage the function of the protein by decreasing its stability (high confidence prediction scores). This is in line with the notion that mutations occurring within the N terminal domain of TSC1 (50-224aa) hinder protein stability and are causative of constitutive mTORC1 signaling [26], [27]. The second family (Pt 3-4) carried a stop codon mutation at Arg692, predicted to cause the generation of a truncated protein. While the truncated Arg692 TSC1 form was found when the corresponding cDNA was transfected in *Tsc1*KO mouse embryonic fibroblasts (MEF) (Figure S1A, B), it was undetectable in patient's cells (Figure S1C). In a previous study, the truncated protein proved able to bind TSC2 and inhibit mTORC1, although with reduced efficiency when compared to unmutated TSC1 [26].

**Figure 1:**
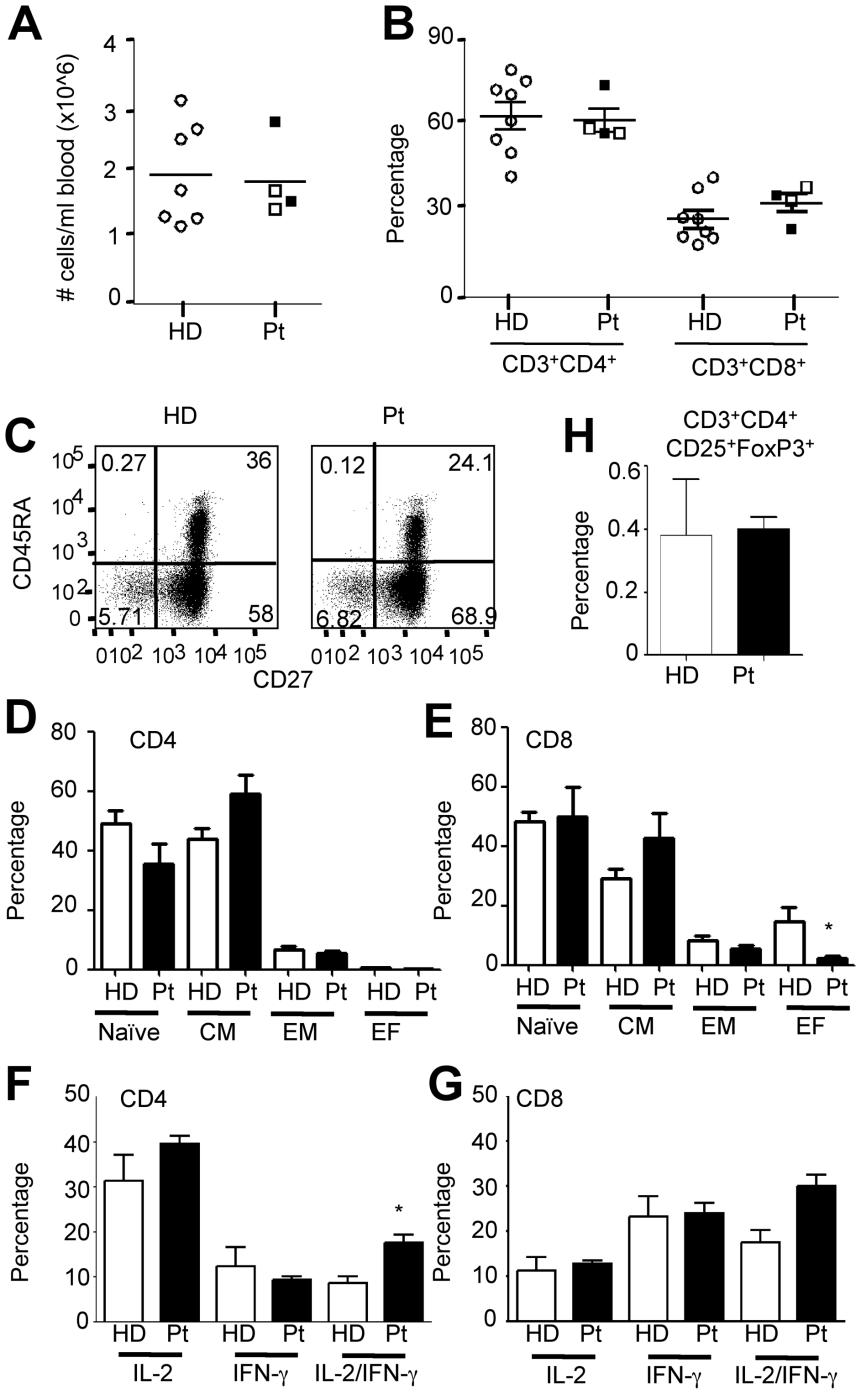
TSC subjects with germline mutation of *TSC1* reveal proper representation of CD4 and CD8 mature T cell subsets. PBMC from healthy donors and TSC subjects were isolated by density gradient centrifugation, counted and surface stained with anti-CD3, CD4, CD8, CD45RA and CD27 mAb and analyzed by FACS. A–B) Viable cell counts per ml of blood and the frequency of CD4^+^ and CD8^+^ cells within CD3^+^ lymphocytes is depicted for individual subjects. Open squares refers to Pt1 and 2 carrying the Leu129Pro mutation, black squares refers to Pt3 and 4 carrying a stop codon mutation in the position of Arg692. C–E) Distribution of naïve (Naïve, CD45RA^high^CD27^high^), central memory (CM, CD45RA^low^CD27^high^), effector memory (EM, CD45RA^low^CD27^low^) and terminally differentiated effectors (EF, CD45RA^high^CD27^low^) within CD3^+^CD4^+^ (C, D) or CD3^+^CD8^+^ (E) cells is depicted. F–G) Freshly isolated PBMC were stimulated for 4 h with PMA and Ionomycin. Expression of IL-2 and IFN-γ was determined by intracellular cytokine staining. Results depict the frequency of cytokine secreting cells after gating on CD3^+^CD4^+^ (F) or CD3^+^CD8^+^ (G) cells (2 HD and Pt3, 4). Cytokine production was also analyzed after a 5d CD3/CD28-driven culture for 8 HD and the 4 TSC1 Pts with no differences (not shown). H) The frequency of CD3^+^CD4^+^CD25^+^FoxP3^+^ cells is shown. Data depicted in D, E, H reflect the mean ± SEM of 8 HD and 4 TSC1 Pts. Statistical significance was evaluated by unpaired two-tailed Student t-Test (A–B, F–H) or Mann-Whitney test (D–E).

To define T cell subsets distribution and phenotype, we derived PBMC from TSC subjects and healthy sex and age-matched donors. Cell counts and flow cytometry analyses revealed comparable number of mononuclear cells (Figure 1A) and representation of CD3^+^CD4^+^ and CD3^+^CD8^+^ T cell subsets (Figure 1B). In addition, recent thymic emigrants (CD45RA^+^CD62L^high^CD31^+^, not shown) and mature T cells with naïve (Naïve: CD45RA^+^ CD27^high^), central memory (CM: CD45RA^−^ CD27^high^), effector memory (EM: CD45RA^−^ CD27^low^) and terminal differentiated effector (EF: CD45RA^+^ CD27^low^) phenotypes were all similarly represented among both CD4^+^ and CD8^+^ T cell subsets of TSC subjects (Figure 1C, D, E), with the exception of terminal differentiated CD8^+^ effector cells, which appeared to be significantly reduced in frequency in TSC1 subjects. To further address possible differences in T cell differentiation, we analyzed effector-like or T regulatory phenotypes by intracellular staining and *ex vivo* T cell responses to specific antigens and polyclonal stimuli by ELISPOT. Results depicted in Figure 1F, G indicate that a similar fraction of CD4 and CD8 T cell subsets producing IL-2 and IFN-γ among PBMC of healthy donors and TSC patients, with only a slight enrichment of IL-2^+^/IFN-γ^+^ cells among CD4^+^ cells of TSC subjects. Following a 5d culture in non-polarizing conditions, however, differences were no longer detectable, and IFN-γ^+^ cells were similarly enriched in all samples (not shown). Less than 5% of the cells produced IL-4 *ex vivo* or upon *in vitro* cell expansion (not shown). Moreover, Treg cells, defined as CD3^+^CD4^+^CD25^+^FoxP3^+^ cells, appeared properly represented (Figure 1H). Thus, overall, at the polyclonal level, T cell subset representation and differentiation appeared to be largely unaffected in TSC subjects with monoallelic *TSC1* mutations, and subtle differences in effector markers did not reveal consistent and possibly attributable to variability among donors.

Finally, to measure Ag-recall responses we adopted HLA-A2-restricted Epstein Barr-virus (EBV) determinants. Out of the four TSC subjects, one was found to express HLA-A2 (Figure S2A). In recall assays, we found EBV-specific T cell responses to be comparable to those of the healthy control (Figure S2B). In addition, PHA and third party allo-responses of TSC subjects revealed similar to those found in a representative healthy donor (Figure S2B). Thus, TSC subjects with known *TSC1* monoallelic germline mutations revealed a normal representation of mature T cell subsets (T naïve, T_CM_, T_EM_, T_eff_; Treg) in the peripheral blood, which appeared capable of memory recall responses.

### TSC/mTOR-dependent signaling, proliferation and survival are preserved in T cells from TSC individuals

**Figure 2:**
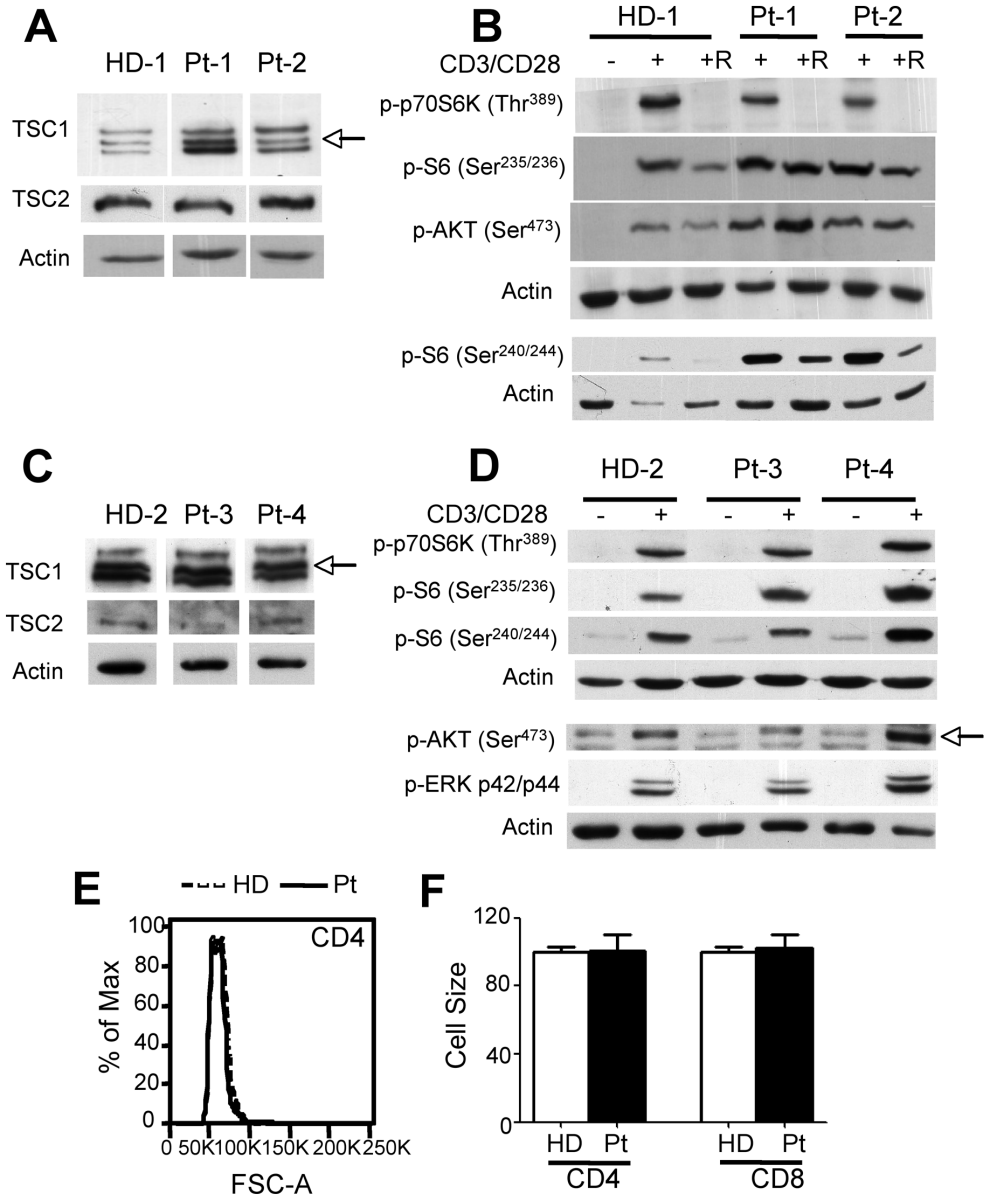
mTOR-dependent signaling is preserved in human T cells with monoallelic germline *TSC1* mutations. A–D) Human CD3^+^ lines derived from healthy donors (HD) and TSC1 subjects (Pt1-4) (see methods) were left untreated (-) or stimulated with anti-CD3 and CD28 mAb for 30 min (+). Where indicated cells were pretreated for 30 min with Rapamycin and then stimulated with anti-CD3/CD28 mAb (+R). Cell extracts were analyzed with the indicated antibody. Arrows indicate the specific bands. E-F) Cell size was determined in fresh PBMC isolated from 8 HD and 4 TSC1 Pts by FACS and is depicted after gating on CD4^+^ or CD8^+^ cells. In E a representative overlay is depicted, while data in F represent the average cell size ± SEM.

**Figure 3:**
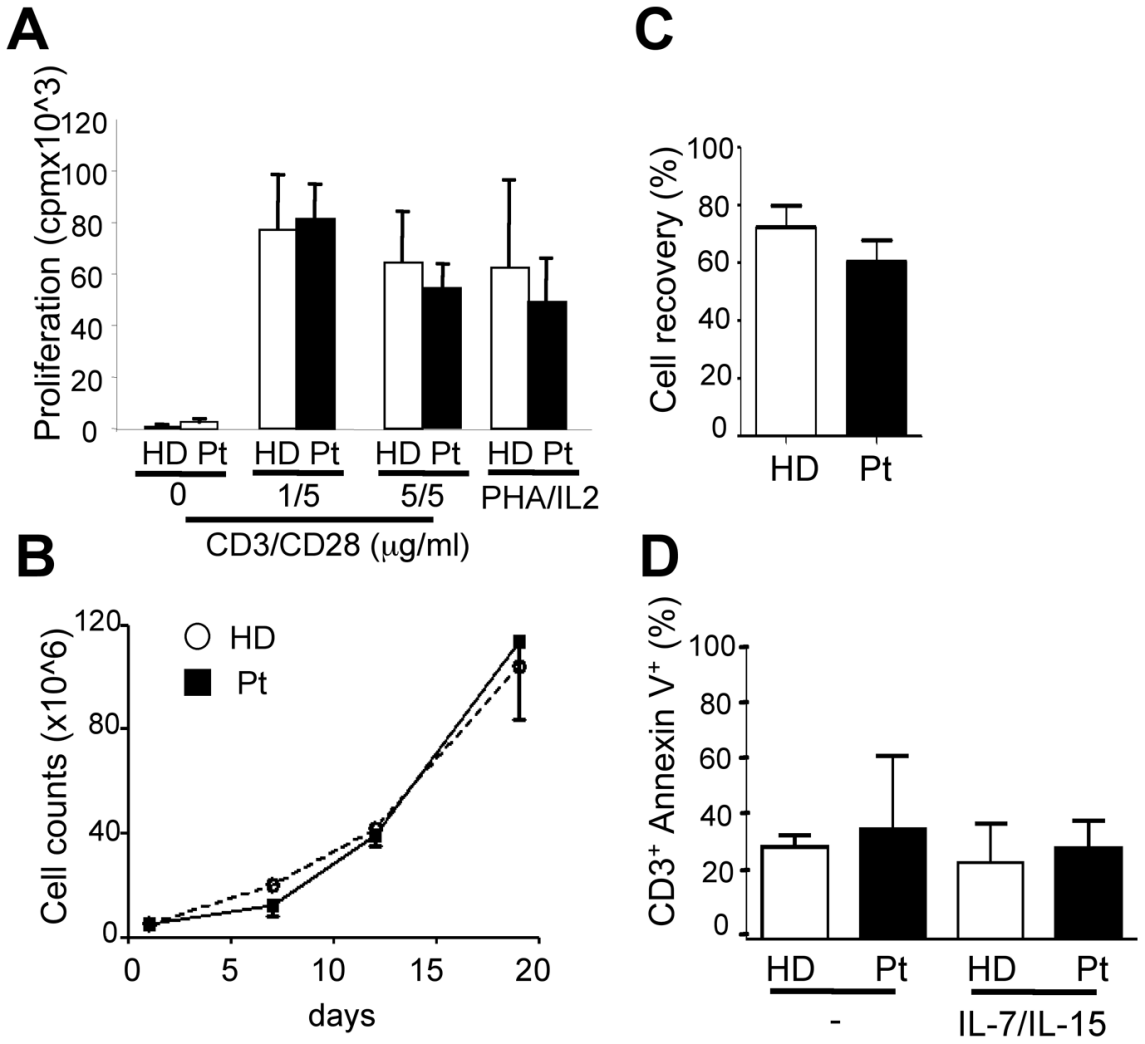
T cells from TSC subjects proliferate and survive to similar extents in response to mitogenic signals. A) Freshly isolated PBMC of four healthy donors (HD, white bars) and four TSC subjects (Pt, black bars) were left unstimulated (0) or activated on plate-coated anti-CD3 and anti-CD28 mAb or PHA and IL-2 for 4 days. Proliferation was assessed by a 18 h-^3^H-thymidine incorporation assay. Data depict mean ^3^H-thymidine incorporation ± SEM. B) freshly isolated PBMC (1 HD, 2 TSC1 Pts) were activated on anti-CD3/CD28 coated beads for 6 days. Thereafter, T cells were isolated and maintained in culture with IL-7 and IL-15 (see methods). At the indicated times, viable counts were obtained. Data are depicted as fold expansion over input and represent one out of two independent determinations. C) Freshly isolated frozen PBMC were thawed and cultured overnight in complete medium. Viable cells were counted. Data are expressed as percentage of input and represent the mean of 10 HD and 4 TSC1 Pts (± SEM) analyzed in independent experiments. D) Frozen CD3^+^ cell lines obtained from 2 HD and 3 TSC1 Pts were thawed, and cultured (2×10∧6/ml) in the absence (-) or presence of IL-7 and IL-15 (5 ng/ml). After 24 h cells were analyzed by flow cytometry. CD3^+^, Annexin V^+^ cells are depicted.

Next we analyzed TSC/mTOR-dependent signaling, cell size, mitogen-induced proliferation and survival in T cells from TSC subjects. To obtain sufficient number of cells, CD3^+^ T cell lines were obtained by stimulating PBMC with anti-CD3/CD28 coated beads for 6 days and then culturing the cells for additional 2 weeks in the presence of IL-7/IL-15, as previously described [28]. To evaluate TSC/mTOR-dependent signaling, cells were left untreated or stimulated with plate-bound anti-CD3/CD28 mAbs in the absence or in the presence of Rapamycin, and analyzed by Western blot analyses. Results indicate that TSC1 and TSC2 levels in T cell lines from healthy donors and TSC subjects, although somehow variable when quantified in independent determinations, appeared to be comparable (Figure 2A, C and Figure S3). Likewise, phosphorylation of p70S6K, S6, and of AKT (Ser473), typical readouts of mTORC1 and mTORC2 activities, respectively, were mostly undetectable in resting cells, and induced to comparable extents in response to CD3/CD28 stimulation (Figure 2B, D and Figure S3). As expected, while CD3/CD28-induced phosphorylation of p70S6K and S6 were acutely sensitive to Rapamycin, this was not the case for AKT on Ser473. In addition, also ERK phosphorylation was similarly regulated in CD3^+^ lines from TSC subjects and healthy donors (Figure 2D). Thus, as also found in mouse T cells with monoallelic *Tsc1* inactivation (Figure S4, see below), one *Tsc1* allele appeared to be sufficient for TSC1 and TSC2 expression and controlled mTORC signaling. In accordance, CD4^+^ and CD8^+^ lymphocytes within PBMC of healthy donors and TSC subjects showed comparable size (Figure 2E, F) and proliferated to similar extents in response to CD3/CD28 antibodies (Figure 3A and B). Biallelic deletion of *Tsc1* in mouse T cells hinders cells survival [21], [22], [23], [24]. We found that cell recovery after CD3/CD28-stimulation (Figure 3B) or after a “freeze and thaw” cycle and overnight rest in plain medium was comparable in TSC subjects and healthy donors cultures (Figure 3C). Likewise, cultures of CD3^+^ T cell lines maintained in complete medium without or with IL-7/15 revealed similar frequencies of Annexin V^+^ cells (Figure 3D). Thus, survival potential of T cells with monoallelic *TSC1* germline mutations appeared comparable to those from healthy donors. Of note, these results were consistent with those obtained with T cells from mice with monoallelic T-cell restricted inactivation of *Tsc1* (Figure S4). While control mice and mice with T-lineage-restricted monoallelic *Tsc1* deletion (Hz) had comparable number of mature CD4 T cells, mice with biallelic *Tsc1* deletion (KO) showed reduced number of mature T cells (Figure S4A). This correlated with the observation that while control and *Tsc1* heterozygous T cells revealed normal TSC1/2 levels and proper control of mTORC1/2 signaling, as well as homeostasis and survival, T cells with biallelic *Tsc1* inactivation showed unbalanced mTORC1/mTORC2-controlled signaling. Indeed, while mTORC1-controlled phosphorylation of p70S6K and S6 was augmented, mTORC2-controlled AKT and FoxO phosphorylation was diminished (Figure S4C, D). This correlated with increased FoxO-targets expression, loss of mitochondrial potential and occurrence of apoptosis (Figure S4E-G). The latter appeared insensitive to Rapamycin (Figure S4H).

Thus, results generated with primary human and mouse T cells with monoallelic *TSC1* inactivation support the notion that one functional *TSC1* allele preserves proper TSC1/2 expression and mTOR signaling, as well as normal proliferation, differentiation and survival of primary lymphocytes. In contrast severe TSC1 down-regulation results detrimental to T cell survival.

### *TSC1* silencing hinders mTORC2/AKT/FoxO signaling, mitochondrial functions and survival of human T cells

**Figure 4:**
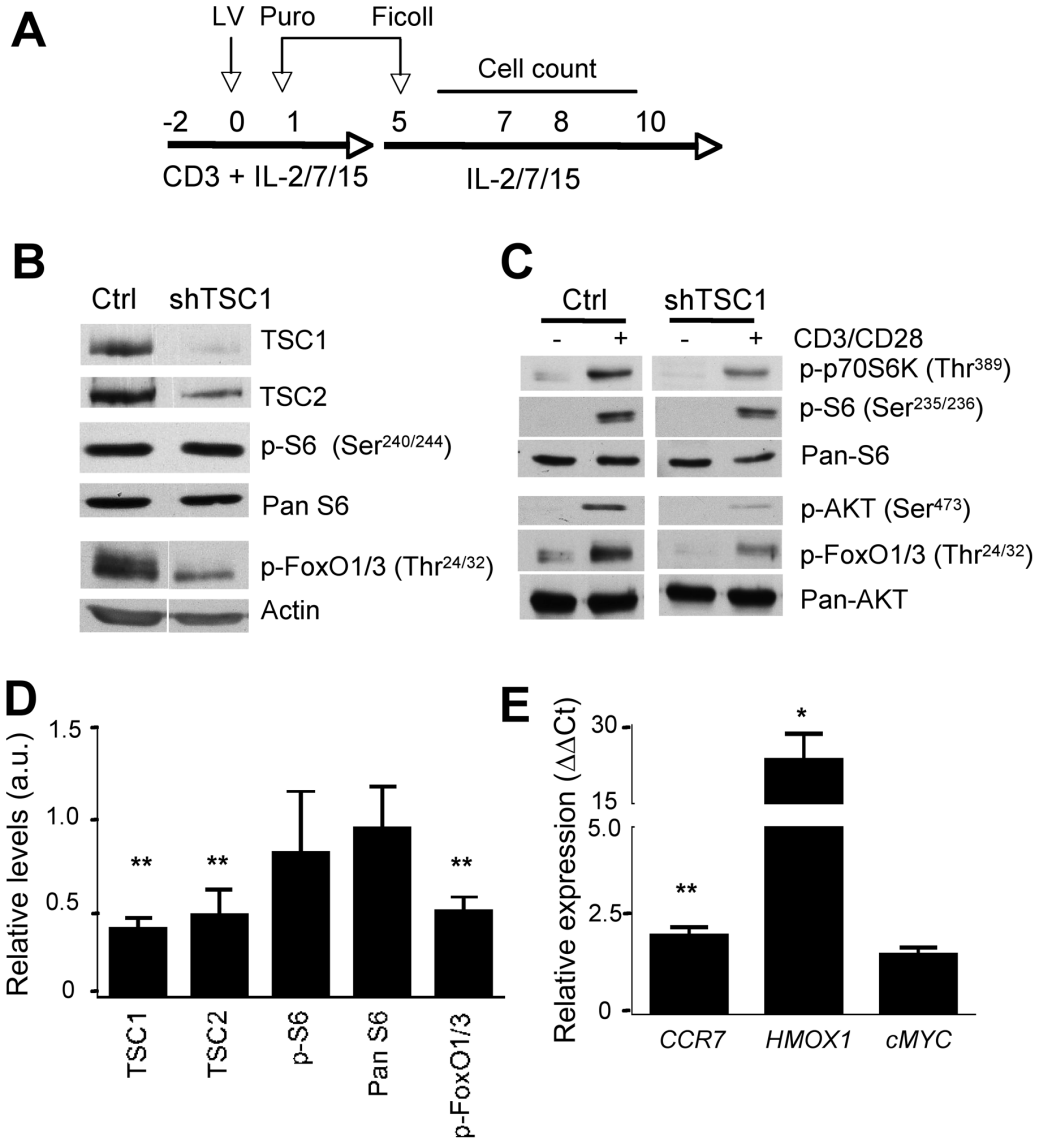
shRNA-assisted TSC1 knock down hinders mTORC2 dependent regulation of FoxO1/3. Human PBMC from 10 independent healthy subjects were activated with anti-CD3 mAb in the presence of IL-2, IL-7 and IL-15 for 48 hours, and transduced with lentiviral vectors overnight (shTSC1). A day after cells were selected for additional 4 days in Puromycin. By then, no viable cells could be recovered from untransduced Puromycin-treated cells. Viable cells in Ctrl and shTSC1 cultures were separated on a Ficoll gradient, counted and re-plated in fresh complete medium supplied with IL-2, IL-7 and IL-15. A) Schematic representation of T cell transduction/selection. B–E) Following viable cell selection at day 5, cells were analyzed by WB (B–D) and real time PCR (E). In B, D cells were left untreated, while in C they were left untreated (-) or stimulated (+) in the presence of anti-CD3 (2 ug/ml) and anti-CD28 (5 ug/ml) coated mAb for 30 min, lysed and analyzed by WB. Representative images (B–C) and quantification (D) of 3-6 independent determinations are depicted. Relative levels (± SEM) refer to expression of given proteins normalized to Actin first, and then to control cells. In E, expression of selected FoxO target genes was normalized to the housekeeping gene (*GAPDH*) and expressed relatively to control cells by the ΔΔCt method. Results depict 4–5 independent determinations. Statistical significance was evaluated by paired, two-tailed Student t-Test.

**Figure 5:**
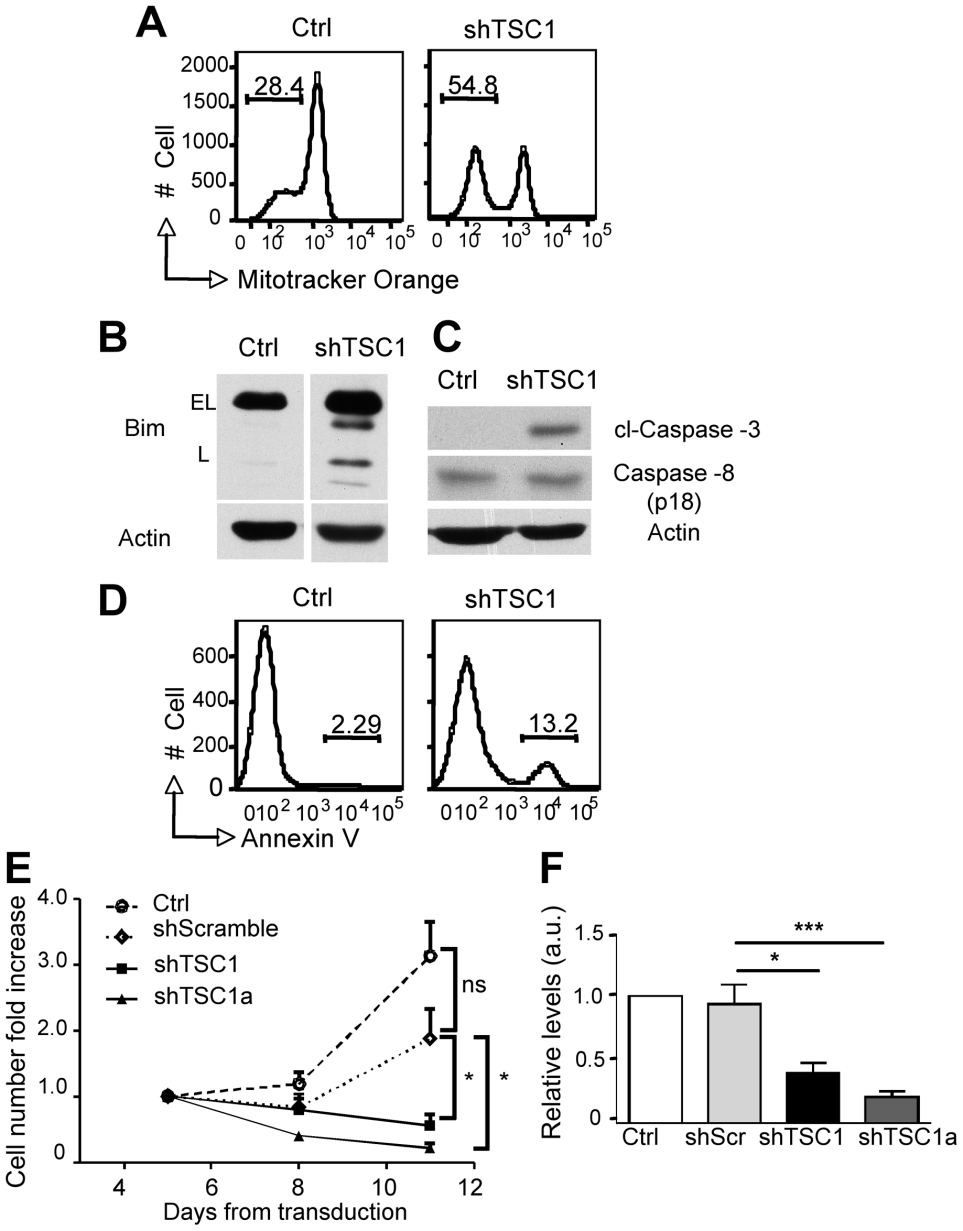
shRNA-assisted TSC1 knock down hinders survival of primary human T cells. shTSC1 cells were obtained by lentiviral-mediated infection and Puromycin selection as indicated in Figure 4A. Control cells and TSC1 knock down cells were harvested at day 8 (three days after Ficoll) and stained with the Mitotracker Orange dye, or with Annexin V and analyzed by FACS (A and D, respectively), or lysed and analyzed by WB (B–C). In A and D, histograms are representative of 4 independent determinations. E) Cell viability of control cells and cells infected with shScrambled, shTSC1 and shTSC1a-recombinant lentiviral vectors was determined over time by Trypan blue counts. Data are depicted as fold changes over the input, and are representative of mean ± SEM of four independently performed experiments. Statistical significance at day 11 was evaluated by unpaired two-tailed Student t-Test. F) TSC1 levels ± SEM of Ctr, shScrambled, shTSC1 and shTSC1a-infected cells were analyzed by WB analysis in four independent determinations. Statistical significance was evaluated by unpaired two-tailed Student t-Test.

To mimic the loss of *TSC1* gene function, possibly occurring in TSC subjects, we infected PBMC of healthy donors with lentiviral vectors encoding for scrambled or *TSC1* shRNA (a schematic representation of T cell transduction and selection is provided in Figure 4A). Following activation by OKT3 and IL-2/7/15 for 2 days, cells were transduced and then cultured for additional 5 days with IL-2/7/15 in the presence of Puromycin. By the time of analysis, up to 95% of the cells expressed CD3, with CD4^+^ and CD8^+^ T cells equally represented (not shown). Western blot analysis performed on unfractionated CD3^+^ cells indicated that both TSC1 and TSC2 levels were drastically reduced in shTSC1 cultures (Figure 4B and D). In spite of this, mTORC1 did not seem to be differently active. Indeed, phosphorylation of p70S6K in Thr^389^ and of S6 in Ser^235/236^ were undetectable in the absence of further stimulation, and yet inducible to comparable extents after CD3/CD28 stimulation in both control and shTSC1 cells (Figure 4C). In contrast, S6 phosphorylation in Ser^240/244^ appeared to be constitutive in control and shTSC1 cells, likely reflecting active mTORC1 because of the cells being cultured in gamma-chain cytokine-enriched medium (Figure 4B). We speculate that S6 phosphorylation in Ser^240/244^ might be easier to detect than that of p70S6K because of different sensitivities of the antibodies, relative phosphorylation levels or signal amplification (the former being downstream to the latter). Differently, mTORC2 appeared defective, as indicated by the lower phosphorylation of AKT and FoxO1/3 in shTSC1 cells either at basal level (Figure 4B, D) or following CD3/CD28 activation (Figure 4C). Reduced phosphorylation of FoxO was mirrored by a higher expression of several of FoxO targets (Figure 4E), and a higher fraction of the cells showing a low mitochondrial membrane potential (Figure 5A), recapitulating results obtained with *Tsc1* KO mouse T cells (Figure S4). The lower mitochondrial potential, together with Bim up-regulation (Figure 5B), likely accounted for the activation of the intrinsic apoptotic pathway (Figure 5C), and the presence of a higher fraction of Annexin V^+^ cells within shTSC1 cultures (Figure 5D). Accordingly, while control and scrambled shRNA infected cells persisted over time in culture, shTSC1 failed to survive (Figure 5E). The use of a second shRNA able to downregulate TSC1 (shTSC1a; Figure 5F) similarly impaired T cell survival (Figure 5E). Thus, TSC1 down-regulation hinders proper mTORC2 control, and favors death of primary human T cells.

Since hamartomas are frequently observed in brain, kidney and skin, but have not been associated with hematological malignancies, we investigated the effect of TSC1 down-regulation in transformed Jurkat cells (Figure S5A). Also in this case, TSC1 down-regulation reduced TSC2 levels and AKT/FoxO1/3 phosphorylation (Figure S5B, C). This correlated with an increase of FoxO's transcriptional activity (Figure S5D), the decrease in mitochondrial membrane potential (Figure S6A), the activation of the intrinsic apoptotic pathway (Figure S6B), a higher representation of Annexin V^+^ cells (Figure S6C) and the loss of cell recovery (Figure S6D).

Thus, down-regulation of *TSC1* levels leads to deregulated mTOR signaling and to apoptotic cell death, in both primary and transformed human T cells.

## Discussion

Before this study, whether TSC subjects with inherited germline mutations of *TSC1* would preserve proper development and homeostasis of mature T lymphocyte and/or whether clinical manifestation of TSC had an impact on immune competence of affected subjects was unknown. Although TSC does not appear to be a disease directly involving the immune system, the possibility that a weaker immune response could contribute to the multiple occurrences of hamartomas remained to be formally proven. We analyzed the immune competence by characterizing PBMC of two TSC Italian families, carrying distinct monoallelic *TSC1* germline mutations identified in hamartomas in various anatomical locations. While transfection of the mutated cDNA elicited detectable expression of both the L129P and the Arg692 mutants, the Arg692 truncated protein could not be detected in T cells from TSC subjects. While we are in the process of testing possible dominant negative effects due to co expression of the wild type and the mutant allele, overall TSC1, TSC2 levels and mTORC1/2-dependent signaling in TSC T cells did not significantly differ from those found in T cells from healthy donors. Accordingly, we found proper representation of mature T and B cell subsets and also comparable frequencies of effector T cell subsets (including recent thymic emigrants, naïve, central memory, effector memory and terminal differentiated effector mature T cells) and of T regulatory subsets. While we found a small but significant increase in the frequency of CD4^+^IL-2/IFN-γ^+^ cells (Pt3, 4), this was not confirmed upon T cell culture of TSC cells (Pt1-4), and was not paralleled by a skewing in the effector or effector memory subsets. In addition, it was not observed in mouse T cells with heterozygous *Tsc1* expression (discussed later on). Thus, results indicate that the observed difference might not be of clinical relevance. Nevertheless, subtle differences might exist, and will be further addressed as soon as more TSC subjects will become available. In addition, cell survival and T cell responses to TCR engaging ligands of T cells from TSC subjects were comparable to those elicited in healthy controls, indicating that T cells with monoallelic germline mutations in *TSC1* appear indistinguishable from wild type ones. This was explained by the finding that TSC1, TSC2 levels and mTORC1/2-dependent signaling did not significantly differ from those found in healthy donors. Thus, inheritance of one functional *TSC1* allele appears sufficient to preserve T cell development and homeostasis in humans. Although the number of patients analyzed remains small, and signaling data suffer of some variability, likely due to the use of T cell lines, we feel confident in concluding that Leu129Pro and Arg692 monoallelic *TSC1* mutations is not sufficient to alter TSC1 levels and mTORC signaling in human T cells, as also supported by data obtained with mouse T cells with heterozygous *TSC1* inactivation (Figure S4). We obtained these mice concurrently with other groups [21], [22], [23], [24] and characterized in details the representation and function of heterozygous T cells. Results indicate that one functional *Tsc1* allele enables normal thymocytes development and the generation of mature T cells, which are phenotypically and functionally indistinguishable from wild-type cells, supporting the conclusion reached with TSC patients. Thus, we believe that it is unlikely that more subtle differences would be found by the analysis of more TSC subjects with different *TSC1* mutations.

In contrast with T cells with monoallelic germline *TSC1* mutations, human T cells with shRNA-assisted TSC1 down-regulation revealed reduced TSC1 (and TSC2) levels, deregulated mTORC2-dependent signaling and impaired survival. This once again was consistent with the results obtained with mouse T cells with biallelic inactivation of *Tsc1*, which resulted in mTORC2 down-regulation and in peripheral T cell lymphopenia, due to the higher propensity of *Tsc1*KO T cells to undergo apoptotic cell death. Thus, both in mouse and human primary T cells, and of note also in human transformed T cells, significant TSC1 down-regulation reveals incompatible with lymphocyte survival.

Of note, differently with *Tsc1*KO mouse T cells, shTSC1 human T cells did not reveal augmented mTORC1 signaling or enlarged cell size. While phosphorylation of p70S6K and of S6 in Ser^235/236^ required TCR/CD28 stimulation to become detectable in rested CD3^+^ T cells, S6 phosphorylation in Ser^240/244^ was found to be constitutive in both control and shTSC1 cells, supporting mTORC1 being active. We also looked at 4EBP-1, but could not obtain convincing evidence for this protein to be differentially regulated. We speculate that differences between mouse and human T cells with respect to S6 phosphorylation in Ser^235/236^ and Ser^240/244^ could be attributed to the phenotype of the cells (naïve versus memory) and dependence on mTORC1 signaling, or to a different regulation of ERK and mTOR. Nevertheless, given the fact that Rapamycin could not rescue survival of either mouse (Figure S4H), or human (not shown) T cells lacking TSC1, we believe that differences in mTORC1 regulation are secondary to the deregulation of mTORC2, which instead is central to reduced cell survival.

The mechanism by which biallelic *Tsc1* inactivation leads to T cell death has been previously debated in mice [21], [22], [23], [24]. In our study, the propensity of *Tsc1KO* T cells to undergo apoptosis correlated with improper mTORC2/AKT/FoxO. This favored the expression of several proapoptotic genes, among which the gained expression of *Hmox1* and *Bim* (and defective Bim protein degradation following TCR/CD28 activation) correlated with the loss of mitochondrial membrane potential, the down-regulation of Bcl-2 (not shown), and the induction of the intrinsic apoptotic pathway. Of note, TSC1 knock down in human PBMC and transformed Jurkat cells phenocopied genetic inactivation of *Tsc1* in mouse T cells, and reproduced the mTORC2/AKT/FoxO defects.

Previous studies had linked mTORC2/AKT/FoxO deregulation to the loss of mitochondrial potential [21], [22], [23], [24]. We speculate this to be due to FoxO-induced HO-1 expression. Heme oxygenase (HO) is a microsomal enzyme that catalyzes the degradation of heme to biliverdin, and has been suggested to function as a defense system against oxidative stress [29]. In particular HO-1 is induced in response to stress signals, and contributes to cell survival. Its overexpression in astroglial cultures and in astrocytes promoted mitochondrial damage and macroautophagy [30]. In addition, HO-1, by decreasing heme concentration within the mitochondria, impacts on the mitochondrial electron transporter chain. Given the notion that naïve and memory T cells are highly dependent on oxidative phosphorylation [31], [32], mitochondria dysfunctions might compromise cell survival. We speculate that in the absence of TSC1/TSC2, FoxO1/3-induced HO-1 reduces mitochondrial functionality, and by that promote deregulation of Bim and Bcl-2 and cell death. Nevertheless, additional mechanisms, possibly involving non canonical functions of TSC1/TSC2 [33] might also contribute to lymphocyte cell death when TSC1 levels are substantially reduced.

It is noteworthy that the loss of TSC1 at different stages of maturation results in context-dependent phenotypes [20]. While the loss of TSC1 in cells of non hematopoietic origin appears to provide a selective advantage for the cell, allowing the formation of hamartomas and the accumulation of additional mutation in proto-oncogenes and oncogenes [34], it appears to provide a selective disadvantage to hematopoietic stem cells [20] and T cells, possibly as a result of an attenuated PI3K/AKT signaling, as it was shown for benign tumors [35]. Based on our results, we speculate that the occurrence of a second hit somatic mutation causing TSC1 loss of function would have a detrimental effect at the clonal level and lead to deletion of the mutated cell, rather than leading to its clonal expansion. Whether a similar conclusion could be obtained when analyzing TSC patients carrying TSC2 mutations, which cause more severe disease manifestations than TSC1 mutations [36], is under investigation to fully understand the clinical management of this disease.

## Materials and Methods

### TSC Subjects and PBMC characterization

The study was performed according to the principles expressed in the Declaration of Helsinki, and following approval of the San Raffaele Institutional Ethic Committee (Comitato Etico dell'Ospedale San Raffaele). Participants provided their written informed consent to participate in the study. Male TSC subjects belonging to two independent Italian families (n = 4; age 30–58) were identified based on genetic screening and identification of *TSC1* mutations. The first family (Pt1 and 2) carries an amino acid substitution at Leu129 (Leu129Pro), predicted to damage the function of the protein due to its decreased stability; the second family (Pt3 and 4) carries a stop mutation at Arg692 known to cause the expression of a truncated protein, still able to bind TSC2 and inhibit mTORC1, but with reduced efficiency when compared to unmutated TSC1 [26]. Disease manifestations ranged from ungual manifestations to mild mental retardation. A summary of individual TSC manifestations is depicted in Table S1. Upon signing an informed consent, blood samples were drawn from TSC subjects (n = 4) and age and sex-matched male healthy donors (n = 6-10; age 30-56). PBMC were isolated from blood samples by a centrifugation onto a Ficoll gradient (Lymphoprep) according the manufacture instruction. After washing in PBS, viable cells were counted by Trypan Blue exclusion. Cells were immediately used or expansion and isolation of CD3^+^ T cell lines was performed with ClinEx-Vivo CD3/CD28 beads (Invitrogen) according to [28].

### Generation of T-cell restricted *Tsc1*-hetero and -knockout mice

Animal studies were performed in accordance with EU guidelines and with the approval of the Institutional Ethical Committee (Permit number # 513). All surgery was performed under anesthesia, and all efforts were made to minimize suffering. Mice were bread in the SPF Institutional facility. *Tsc1* floxed mice [37] were crossed with CD4-Cre deleter mice allowing deletion of the gene starting at the DP stage in thymic development. Genotyping was performed using a PCR based approach with primers specific for *Tsc1*: F4536 (5′-AGGAGGCCTCTTCTGCTACC-3′), R4830 (5′-CAGCTCCGACCATGAAGTG-3′) and R6548 (5′-TGGGTCCTGACCTATCTCCTA-3′) and for the Cre recombinase: F (5′-TATATCTTCAGGCGCGCGGT-3′) and R (5′-GCAATCCCCAGAAATGCCAG-3′). Expected size of PCR products are: 300 bp: wt *Tsc1*; 500 bp: floxed *Tsc1*; 400 bp *Tsc1* deleted; 300 bp: *Cre*. Mice with T-lineage restricted conditional *Tsc1* deletion used for functional experiments have been backcrossed onto the C57BL/6N background for at least five generations and analyzed at 12–14 wk of age unless otherwise indicated. In all cases, age-matched wild type (WT) littermates were used as controls. All mice were housed in a specific pathogen-free environment.

### FACS analyses

Freshly isolated human PBMC were surface stained for CD3/CD4/CD8/CD45RA/CD27. For FoxP3 intracellular expression, freshly isolated PBMC were stained with CD3, CD4, CD25 surface markers, and fixed, permeabilized according to eBioscence manufacture protocol and then stained intracellularly with anti-FoxP3 mAb or isotype control. Cytokines production in human cells was evaluated on freshly isolated PBMC after stimulation for 4 hours with PMA (0.05 ug/ml) and Ionomycin (1 ug/ml), of which the last 2 in the presence of Brefeldin A (5 ug/ml). Next, cells were surface stained, fixed and intracellularly stained for IL-2 and IFN-γ. Acquisition and analyses were performed using FACSCanto cytometer (BD Biosciences) and FlowJo Software (Tree Star). Mouse splenocytes were stained with directly conjugated mAb from BioLegend and BD Pharmingen (CD4/CD8/CD44/CD62L). Apoptotic cells were detected by Annexin V (BD Pharmingen) and TO-PRO-3 (Invitrogen) staining. Mitotracker Orange CMTMRos (Invitrogen) was used according to manufacturer instruction to measure mitochondrial membrane potential.

### Western blot

Human CD3^+^ T lines were obtained from fresh PBMC by in vitro stimulation with anti-CD3/CD28 coated beads for 6 days. Cells were then rested for an additional 2 weeks in the presence of IL-7/IL-15 (5 ng/ml) as described [28]. To evaluate TSC/mTOR-dependent signaling cells were either left untreated, or stimulated with plate-bound anti-CD3/CD28 mAb (2 ug/ml and 5 ug/ml, respectively), and analyzed by Western blot analyses. Where indicated (Figure 2A; +R) cells were preincubated with Rapamycin (1 uM; 30 min) and then stimulated on immobilized anti-CD3 and anti-CD28 mAb. Cells were then lysed and analyzed by SDS-PAGE as previously described [38]. Mouse CD4^+^ LN T cells were purified by negative selection using anti-CD8 (clone KT15) and anti-I-A^b-d-q^/I-E^d-k^ (clone M5/114) rat Abs and sheep anti-rat-coated magnetic beads (Dynal Biotech LTD., UK) to a purity of >95%. Antibodies were all from Cell Signaling Technology (Milan, Italy), with the exception of anti-Actin and anti-Caspase 8 mAb, which were from Santa Cruz Biotechnology. Were indicated images were quantified by ImageJ-assisted analyses.

### Real-time PCR

RNA was purified with RNeasy Mini Kit (Qiagen) following manufacturer's instructions. Total RNA was retrotranscribed and analyzed by Real time PCR with TaqMan probes (*Trail*, *Fasl*, *Bcl2l11*(*Bim*), *Hmox1*, *Cd127*, *cMyc*) (Applied Biosystems), SYBRgreen master mix (Applied Biosystems) with *CCR7* (F: GGTGGTGGCTCTCCTTGTCATT and R: TGTCTCCGATGTAATCGTCCGTGA) specific primers or LightCycler master mix (Roche; *HMOX1*-F: GCAACCCGACAGCATGCCCC and R: CAGCGGGGCCCGTACCAGAA; *cMYC*-F: GCCGCCGCTCCGGGCTCTGCTC and R: CTGCTGGTGGTGGGCGGTGTC) using the ABI Prism HT7900 Sequence Detection System (Applied Biosystems) or Light Cycler480 Real-Time PCR System (Roche). Ct values were normalized to the value of the housekeeping gene (mouse: *Tbp*, TaqMan Probes; human: *GAPDH*; Qiagen), according to the ΔΔCt method.

### Thymidine Incorporation Assay

0.2×10^6^ PBMC from healthy donors and TSC subjects were stimulated on plate-coated anti-CD3 (1, 5 ug/ml) and anti-CD28 (5 ug/ml soluble) antibodies, and PHA (1 ug/ml) + IL-2 (100 U/ml) as a positive control. Cells were activated for 96 hours, of which the last 18 hours in the presence of thymidine (1uCi).

### LV production and Transduction

Lentiviral stocks were produced by polyethylenimine-assisted transient transfection of 293T packaging cells with pLKO.1 shTSC1 (RHS3979-9607126) or shTSC1a (RHS3979-9607122) (Thermo Scientific), together with pCMV-dR8.74 and VSV-G/pMD 2.G plasmids (kindly provided by Dr. Vincenzo Calautti, Dulbecco Telethon Institute, c/o Molecular Biotechnology Center, University of Torino). pLKO.1-PGK-puro-CMV-Turbo GFP Positive Control (GFP) and pLKO.1 scrambled shControl [39] were used as comparison. Virus supernatants were collected after 30 h, concentrated by ultracentrifugation, and stored in PBS at -80°C. PBMC from healthy donors and Jurkat T lymphoma cell line were cultured in RPMI1640 medium supplemented with antibiotics, glutamine and 10% FBS. 48 h before infection, PBMC were activated with anti-CD3 (OKT-3 clone, 30 ng/ml) and IL-2 (12 IU/ml) in the presence of IL-7 and IL-15 (5 ng/ml each), as previously described with modifications [28]. 48 h after activation cells were infected in the presence of Polybrene and one day later selected for 4 days on Puromycin (2 ug/ml). By this time all non-infected cells were dead. Viable cells were then separated on a Ficoll gradient and analyzed by WB, qRT-PCR and for survival in the absence of Puromycin. Viable cells were enumerated by Trypan blue counting.

### Statistical analysis

Data were analyzed by one-way ANOVA (Bonferroni's post test), Student t-Test and non-parametric Mann-Whitney test where indicated. Statistical significance: * p<0.05; ** p<0.01; ***p<0.001.

## Supporting Information

Figure S1 **Expression of TSC1 mutated proteins in** ***Tsc1*** **KO MEF and TSC patients.** *Tsc1*KO MEF were transfected with plasmids encoding for WT (TSC1) or mutated forms of TSC1 (L129P and Arg692). Twenty-four hours after transfection cells were lysed and the protein extracts were analyzed by Western blot with polyclonal (A and C) and monoclonal (B) anti-TSC1 Abs (both from Cell Signaling). The asterisks indicate the bands corresponding to either the full-length TSC1 protein (150 kDa) or the truncated form (80 kDa). In C also CD3^+^ cells obtained from T cell lines established from healthy donors and TSC subjects (Pt3-4) were analyzed.
(TIF)

Figure S2 **Polyclonal and Antigen-specific responses are preserved in T cells from TSC subjects.** A) TSC subjects were analyzed for HLA-A2 expression by FACS, as compared to a HLA-A2^+^ and HLA-A2^−^ lines. Pt4 was found to be HLA-A2^+^. Representative histograms are depicted. B) Freshly isolated PBMC from a representative healthy donor (HD) and two TSC subjects (Pt3 and 4) were stimulated overnight with PHA or a HLA-A2-restricted EBV peptide. Pt T cells were also stimulated with irradiated HD cells (Allo). IFN-γ-secreting cells were quantified by ELISPOT.
(TIF)

Figure S3 **mTOR-dependent signaling in human T cells with monoallelic germline** ***TSC1*** **mutations.** Human CD3^+^ lines derived from healthy donors (HD) and TSC1 subjects (Pt1-2) (A) and Pt3-4 (B) were activated as described in Figure 2. Relative expression (TSC1 and TSC2) and phosphorylation of indicated proteins were analyzed by Western blot and quantified by densytometric analysis of at least 2 independent determinations. Statistical significance was analyzed by Student t-Test (TSC1 and TSC2) and One-way ANOVA with Bonferroni's post-test.
(TIF)

Figure S4 **Biallelic loss of** ***Tsc1*** **prompts de-regulated mTOR-dependent signaling, gained FoxO1/3 activity, mitochondrial dysfunction and apoptotic cell death in mouse T cells.** A–B) Single cell suspension of unfractionated lymph node cell pools (auxiliary, bronchially and inguinal) from 12–14 week-old T-lineage restricted *Tsc1* ^+/+^ (WT), *Tsc1* ^+/−^ (Hz), *Tsc1* ^−/−^ (KO) mice were surface-stained and analyzed by FACS. The relative representation of viable CD4^+^ T cells (A) and their relative size (FSC) is depicted (B). The statistical significances was evaluated by One-way ANOVA with Bonferroni's post-test. C-D) CD4^+^ T cells were purified and left untreated (-) or stimulated with anti-CD3 and anti-CD28 mAb for 30 min (CD3/CD28). Relative expression and phosphorylation levels of the indicated targets, assessed by WB analyses, are depicted. Data are representative of 2–5 independent determinations. E) Real time PCR of CD4^+^ T cells. The expression of the indicated FoxO targets, analyzed over 4–7 independent experiments, was first normalized to that of the housekeeping gene (*Tbp*) and next expressed relatively to WT control cells by the ΔΔCt method. F, G). Freshly isolated, unfractionated splenocytes were surface stained and then evaluated for mitochondrial membrane potential with the Mitotracker Orange dye (F) or for apoptosis by Annexin V staining (G). In H, splenocytes were cultured overnight in complete medium in the absence or in the presence of Rapamycin. Cell death was then analysed by FACS by Annexin V staining. Data show mitochondrial membrane potential (F) and cell death (G–H) of CD4^+^ T cell population. Statistical significance was evaluated by two-tailed paired Student t-Test (E) and One-way ANOVA with Bonferroni's post-test (G–H).
(TIF)

Figure S5 **shRNA-assisted TSC1 knock down hinders mTORC2 dependent regulation of FoxO1/3 in transformed Jurkat T cells.** Jurkat T-leukemia cells were transduced by lentiviral-encoding TSC1 shRNA or scrambled control GFP-tagged shRNA. Cells were selected for 4 days in Puromycin. By then, no viable cells could be recovered from untransduced Puromycin-treated cells. Viable transduced cells separated on a Ficoll gradient, counted and re-plated in fresh complete medium. A) Schematic representation of the cell transduction/selection. B–C) Scrambled shRNA (GFP) and TSC1 shRNA-infected cells (shTSC1) were analyzed for WB. In C, densitometry analyses of three experiments are shown. Data were first normalized by Actin, and then expressed relatively to GFP control cells. D) Expression of the indicated FoxO targets was measured by Real time PCR. Data were normalized to the expression of the housekeeping gene (*GAPDH*) and expressed relatively to control cells (GFP) by the ΔΔCt method. Statistical significance was determined by a paired, two-tailed Student t-Test.
(TIF)

Figure S6 **shRNA-assisted TSC1 knock down hinders survival of transformed Jurkat T cells.** GFP and shTSC1 cells were obtained as indicated in Figure S5A. Cells were harvested at day 8 (three days after Ficoll) and stained with the Mitotracker Orange dye (A), or with Annexin V (C) and analyzed by flow cytomerty, or lysed and analyzed by Western blot (B). Histogram overlays (A) and dot plots (C) are representative of 4 and 3 independent experiments, respectively. In B, WB images representative of 2–3 independent determinations are shown. D) Cell viability was determined over time by Trypan blue-assisted counts. Data are depicted as fold changes over input and are representative of one of three independent similar determinations.
(TIF)

Table S1 **Clinical manifestations of TSC in the analyzed sample group.**
(DOC)
