# Correction: Monoallelic Germline *TSC1* Mutations Are Permissive for T Lymphocyte Development and Homeostasis in Tuberous Sclerosis Complex Individuals

**DOI:** 10.1371/journal.pone.0218354

**Published:** 2019-06-07

**Authors:** Karolina Pilipow, Veronica Basso, Nicola Migone, Anna Mondino

After publication of this article [1], concerns were raised about the following:

1. Fig 2D p-p70S6K panel lanes 1 and 2 appear similar to lanes 3 and 4
2. Fig 2A and 2C, the TSC1, TSC2 and Actin panels appear as individual lane/band images
3. Fig 4B, p-FoxO1/2 (Thr 24/32) and Actin panels are comprised of multiple separate blot images
4. Fig 5B, the Bim and Actin panels in Control and shTSC1 lanes appear as individual blot images.
5. Fig 4C, Control and shTSC1 lanes appear as individual blot images.

**Fig 2:**
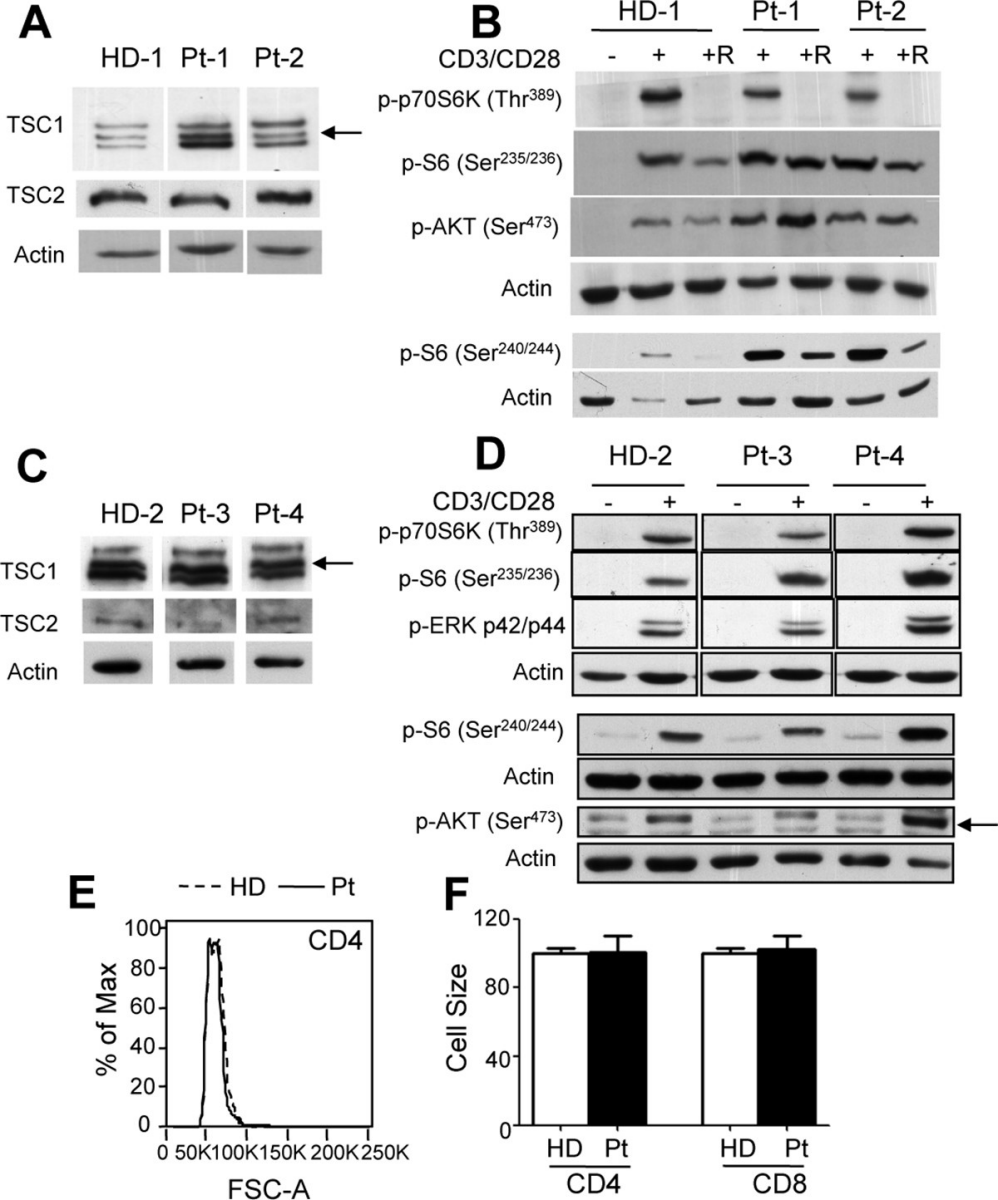
mTOR-dependent signaling is preserved in human T cells with monoallelic germline *TSC1* mutations. A-D) Human CD3^+^ lines derived from healthy donors (HD) and TSC1 subjects (Pt1-4) (see methods) were left untreated (-) or stimulated with anti-CD3 and CD28 mAb for 30 min (+). Where indicated cells were pretreated for 30 min with Rapamycin and then stimulated with anti-CD3/CD28 mAb (+R). Cell extracts were analyzed with the indicated antibody. Arrows indicate the specific bands. E-F) Cell size was determined in fresh PBMC isolated from 8 HD and 4 TSC1 Pts by FACS and is depicted after gating on CD4^+^ or CD8^+^ cells. In E a representative overlay is depicted, while data in F represent the average cell size ± SEM.

The authors have carefully reviewed the data and addressed the concerns as follows:

1) Fig 2D. The authors acknowledge that an error was made in preparing Fig 2D of this article [1]. Data originated from a single gel/blot image, from which some irrelevant lanes were cropped out. In the process of assembling the final figure, lanes 1 and 2 reflecting p-p70S6K were copied twice by mistake. The original data underlying the figure have now been reanalyzed and correctly assembled in revised panel 2D. With this correction, the authors provide an updated Fig 2, along with all raw data underlying edited Fig 2 (S1 File).

2) Fig 2A and 2C. The authors confirm that Fig 2A TSC1, TSC2 and Actin panels depict samples from unstimulated Healthy Donors (HD) and Patients (Pt) cells run on the same gel alongside stimulated ones. Lanes reflecting stimulated cells were cropped out, and only unstimulated samples were assembled in the final figure.

3–4) Figs 4B and 5B. The authors confirm that extracts of control (Ctrl) and TSC1 silenced (shTSC1) cells were run on the same gel. The membranes were cut according to molecular weight and probed with anti-p-FoxO1/2 (Thr 24/32) and Actin mAb. Individual blot images were assembled after cropping out irrelevant lanes.

5) Fig 4C. The authors confirm that control and shTSC1 lanes are individual blot images, corresponding to unstimulated (-) or CD3/CD28-stimulated (+) Ctrl and shTSC1 lanes derive from the same gel. Replicate gels were run and analyzed as follows: p-p70S6K, p-S6, p-FoxO1/3 and Pan-AKT (Gel 1), pAKT (Gel 2) and PanS6 (Gel 3).

Raw data underlying all results reported in the article remain available.

With this Correction, the authors provide the original scans of western blot data underlying Fig 2A–2D in S1 File, the individual-level data underlying panels E and F in S2 File and the uncropped blots underlying Fig 4B, 4C and 5B in S3 File.

A member of PLOS ONE’s Editorial Board confirmed that depicted data are in line with the original message of the manuscript.

The authors apologize for the errors in the published article.

## Supporting information

S1 File Original scan of western blot data depicted in corrected Fig 2. The raw data used to assemble panel A-D of Fig 2 are depicted. Dotted lines reflect the portion of the images that were used to assemble the final panels. No image adjustment was used.
(PDF)

S2 File Individual-level data underlying Fig 2E and 2F. Panel E depicts a FACS overlay representative of data also depicted in panel F as the average cell size ±SEM.
(XLSX)

S3 File Original scan of western blot data depicted in Fig 4B-C, and 5B. The raw data used to assemble panels B and C of Fig 4 and pane B of Fig 5 are depicted. Dotted lines reflect the portion of the images that were used to assemble the final panels. No image adjustment was used.
(PDF)

## References

[1] Pilipow K, Basso V, Migone N, Mondino A (2014) Monoallelic Germline *TSC1* Mutations Are Permissive for T Lymphocyte Development and Homeostasis in Tuberous Sclerosis Complex Individuals. PLoS ONE 9(3): e91952 10.1371/journal.pone.0091952 24633152 PMC3954840

