# Supplementary figures and images for: Monoallelic Germline TSC1 Mutations Are Permissive for T Lymphocyte Development and Homeostasis in Tuberous Sclerosis Complex Individuals
Source: PLoS One. 2014 Mar 14;9(3):e91952. doi: 10.1371/journal.pone.0091952 (PMC3954840; doi:10.1371/journal.pone.0091952)

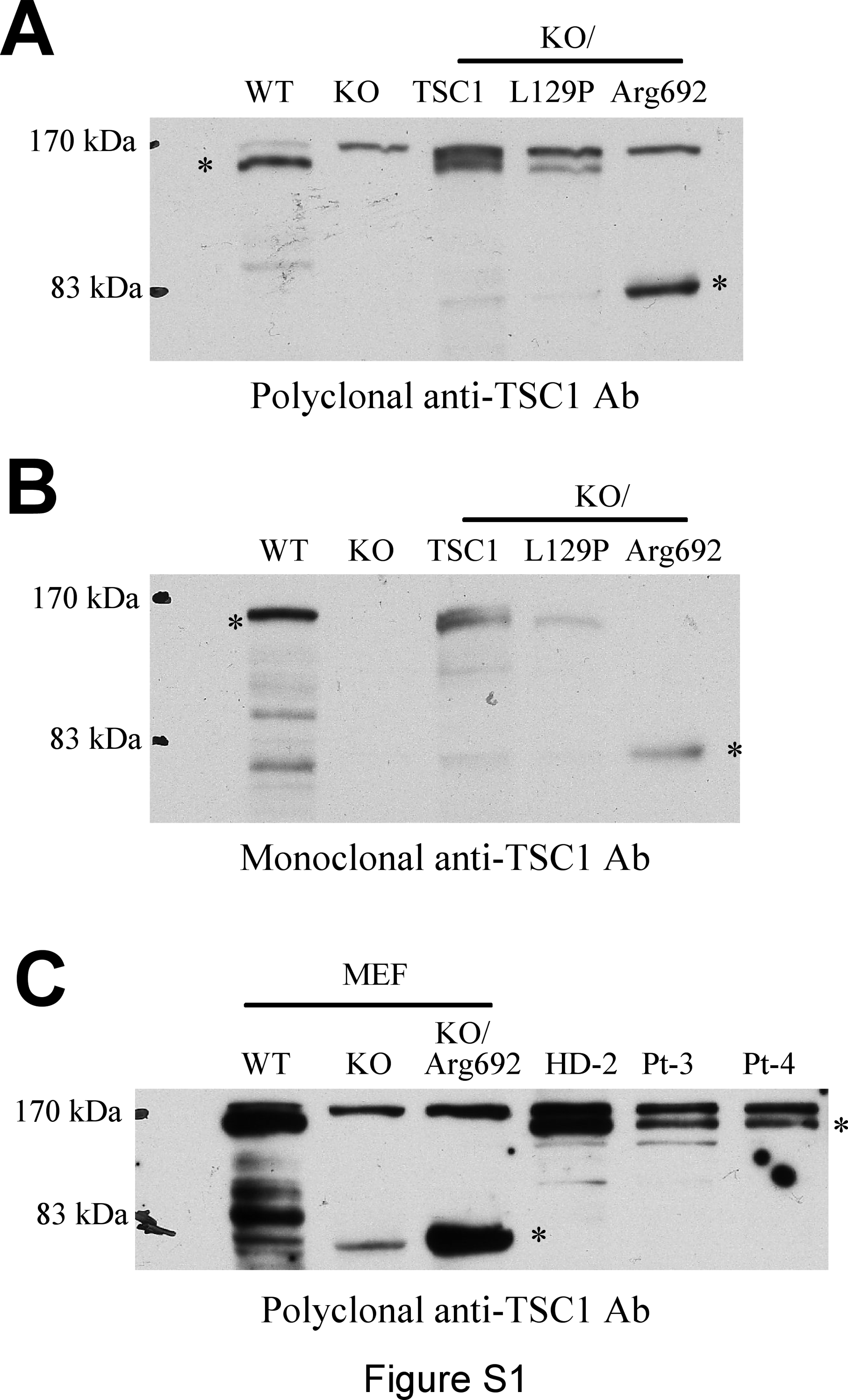

Supplement: Figure S1 — Expression of TSC1 mutated proteins in Tsc1 KO MEF and TSC patients. Tsc1KO MEF were transfected with plasmids encoding for WT (TSC1) or mutated forms of TSC1 (L129P and Arg692). Twenty-four hours after transfection cells were lysed and the protein extracts were analyzed by Western blot with polyclonal (A and C) and monoclonal (B) anti-TSC1 Abs (both from Cell Signaling). The asterisks indicate the bands corresponding to either the full-length TSC1 protein (150 kDa) or the truncated form (80 kDa). In C also CD3+ cells obtained from T cell lines established from healthy donors and TSC subjects (Pt3-4) were analyzed. (TIF) [file pone.0091952.s001.tif]

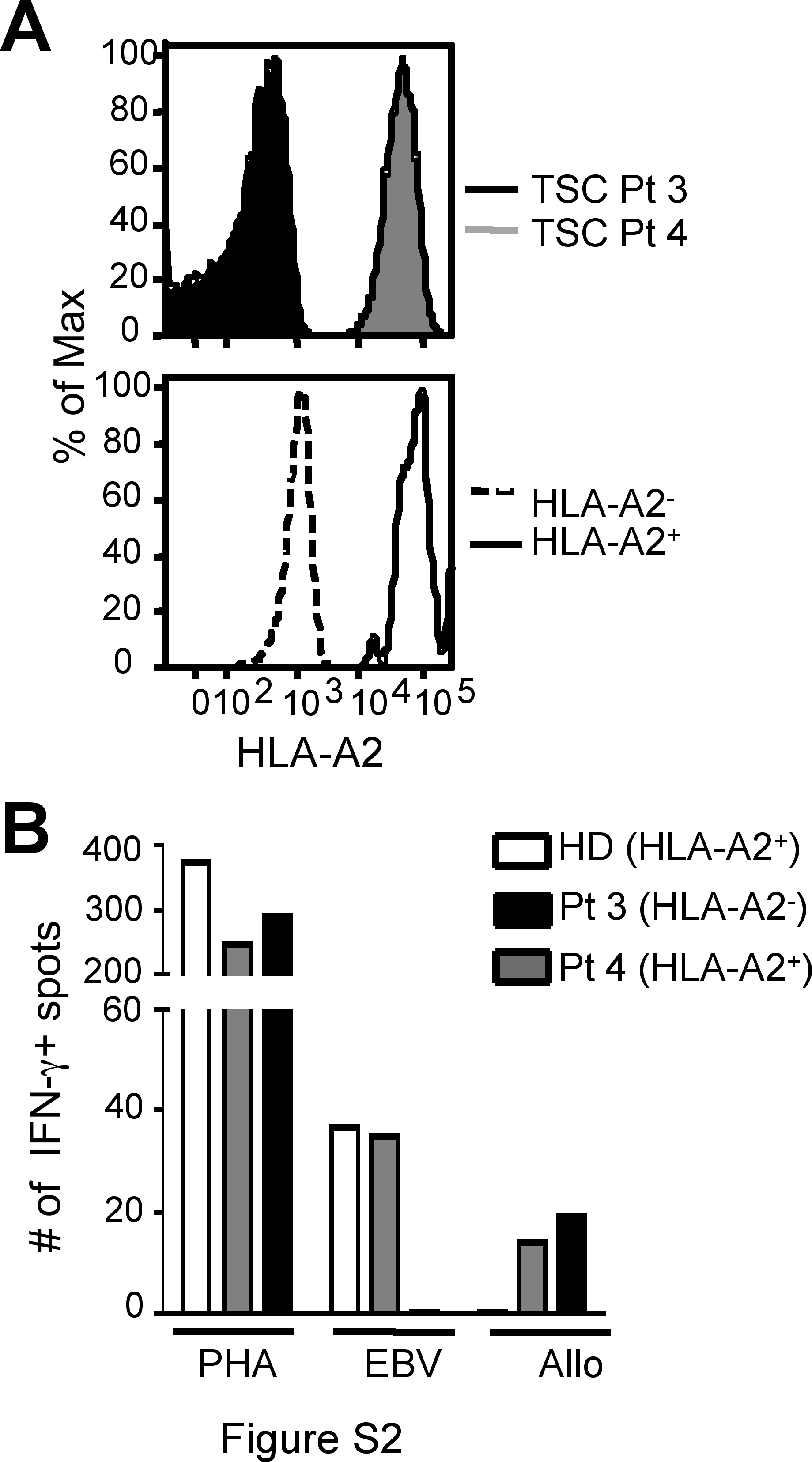

Supplement: Figure S2 — Polyclonal and Antigen-specific responses are preserved in T cells from TSC subjects. A) TSC subjects were analyzed for HLA-A2 expression by FACS, as compared to a HLA-A2+ and HLA-A2− lines. Pt4 was found to be HLA-A2+. Representative histograms are depicted. B) Freshly isolated PBMC from a representative healthy donor (HD) and two TSC subjects (Pt3 and 4) were stimulated overnight with PHA or a HLA-A2-restricted EBV peptide. Pt T cells were also stimulated with irradiated HD cells (Allo). IFN-γ-secreting cells were quantified by ELISPOT. (TIF) [file pone.0091952.s002.tif]

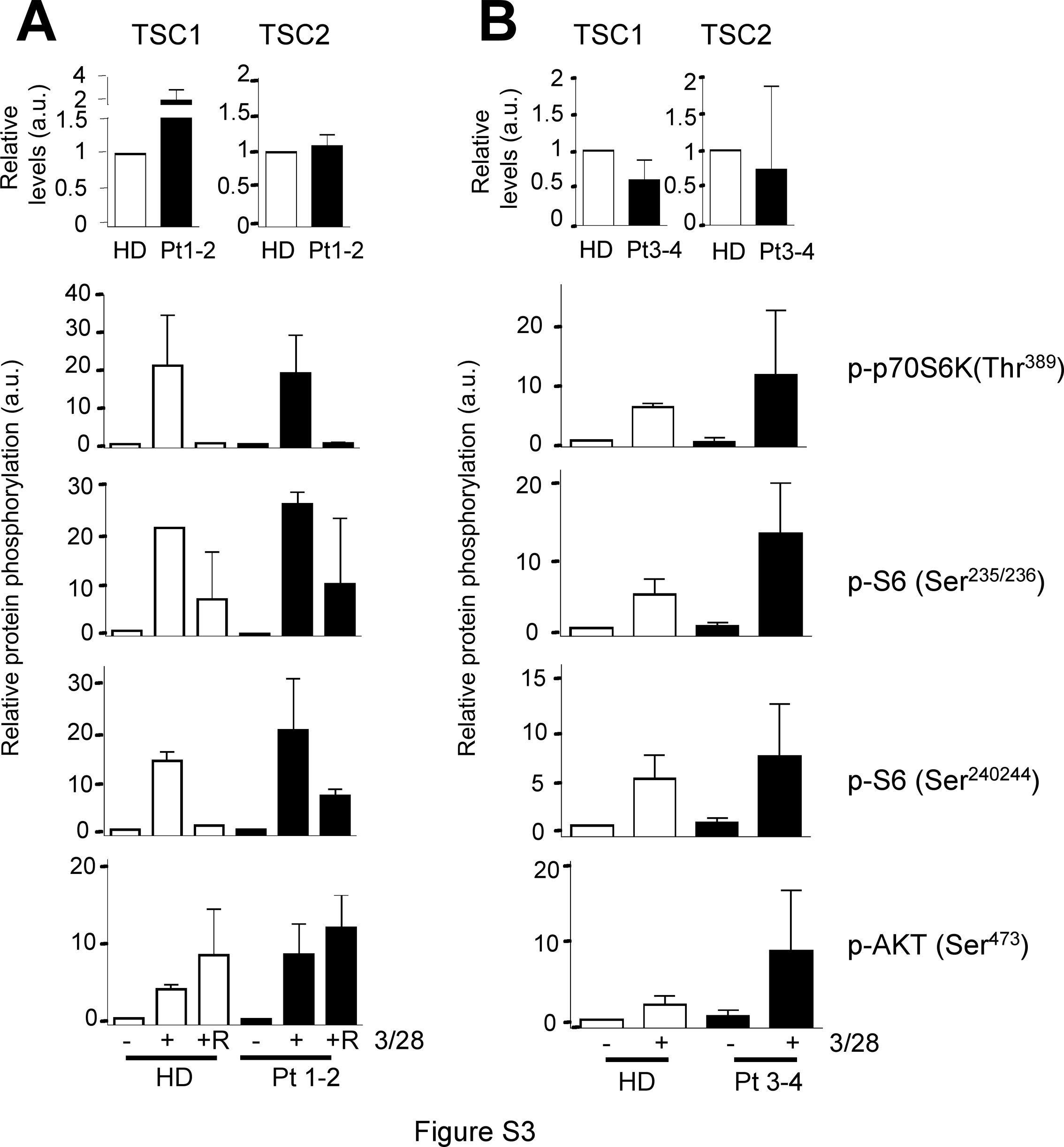

Supplement: Figure S3 — mTOR-dependent signaling in human T cells with monoallelic germline TSC1 mutations. Human CD3+ lines derived from healthy donors (HD) and TSC1 subjects (Pt1-2) (A) and Pt3-4 (B) were activated as described in Figure 2. Relative expression (TSC1 and TSC2) and phosphorylation of indicated proteins were analyzed by Western blot and quantified by densytometric analysis of at least 2 independent determinations. Statistical significance was analyzed by Student t-Test (TSC1 and TSC2) and One-way ANOVA with Bonferroni's post-test. (TIF) [file pone.0091952.s003.tif]

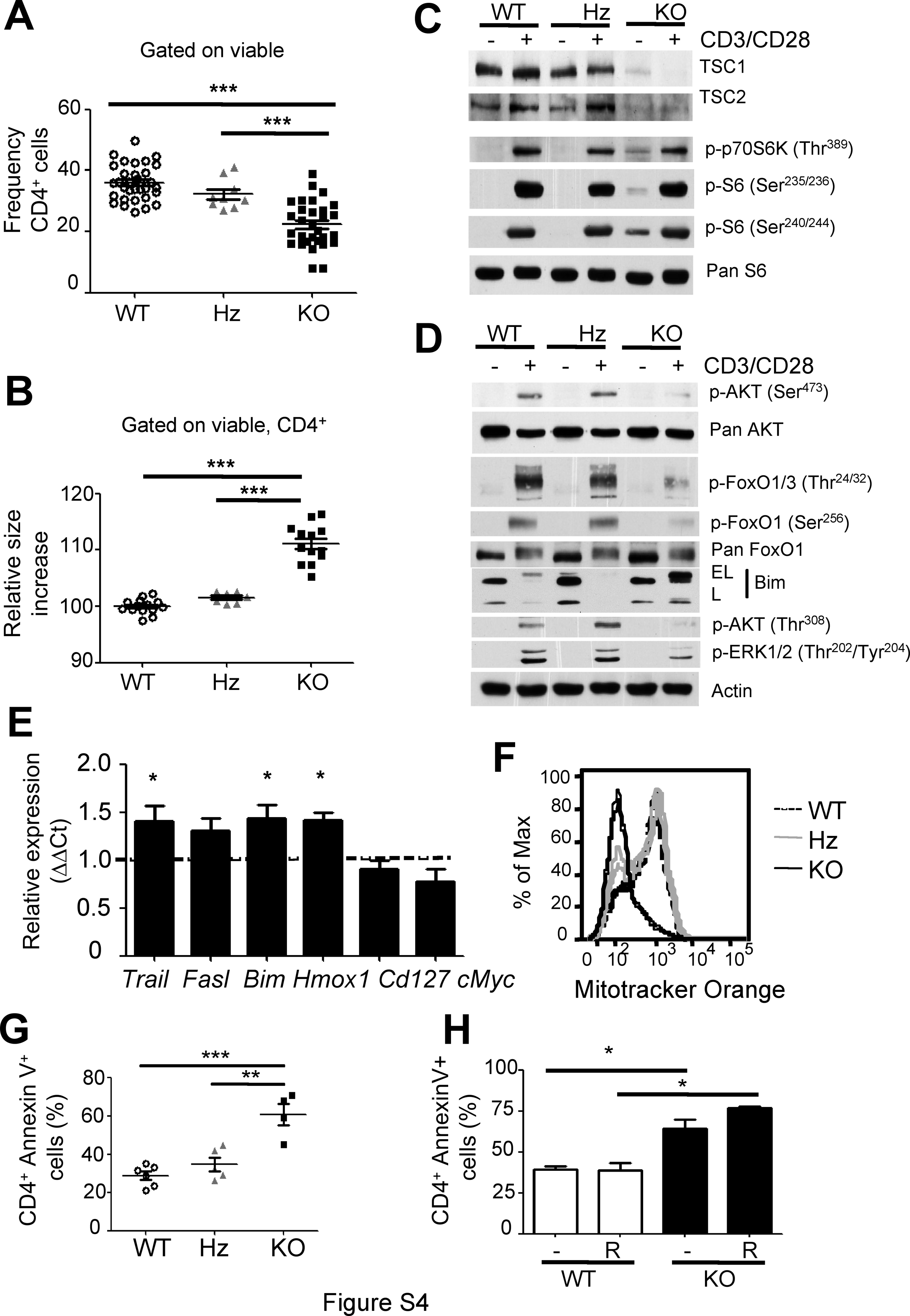

Supplement: Figure S4 — Biallelic loss of Tsc1 prompts de-regulated mTOR-dependent signaling, gained FoxO1/3 activity, mitochondrial dysfunction and apoptotic cell death in mouse T cells. A–B) Single cell suspension of unfractionated lymph node cell pools (auxiliary, bronchially and inguinal) from 12–14 week-old T-lineage restricted Tsc1 +/+ (WT), Tsc1 +/− (Hz), Tsc1 −/− (KO) mice were surface-stained and analyzed by FACS. The relative representation of viable CD4+ T cells (A) and their relative size (FSC) is depicted (B). The statistical significances was evaluated by One-way ANOVA with Bonferroni's post-test. C-D) CD4+ T cells were purified and left untreated (-) or stimulated with anti-CD3 and anti-CD28 mAb for 30 min (CD3/CD28). Relative expression and phosphorylation levels of the indicated targets, assessed by WB analyses, are depicted. Data are representative of 2–5 independent determinations. E) Real time PCR of CD4+ T cells. The expression of the indicated FoxO targets, analyzed over 4–7 independent experiments, was first normalized to that of the housekeeping gene (Tbp) and next expressed relatively to WT control cells by the ΔΔCt method. F, G). Freshly isolated, unfractionated splenocytes were surface stained and then evaluated for mitochondrial membrane potential with the Mitotracker Orange dye (F) or for apoptosis by Annexin V staining (G). In H, splenocytes were cultured overnight in complete medium in the absence or in the presence of Rapamycin. Cell death was then analysed by FACS by Annexin V staining. Data show mitochondrial membrane potential (F) and cell death (G–H) of CD4+ T cell population. Statistical significance was evaluated by two-tailed paired Student t-Test (E) and One-way ANOVA with Bonferroni's post-test (G–H). (TIF) [file pone.0091952.s004.tif]

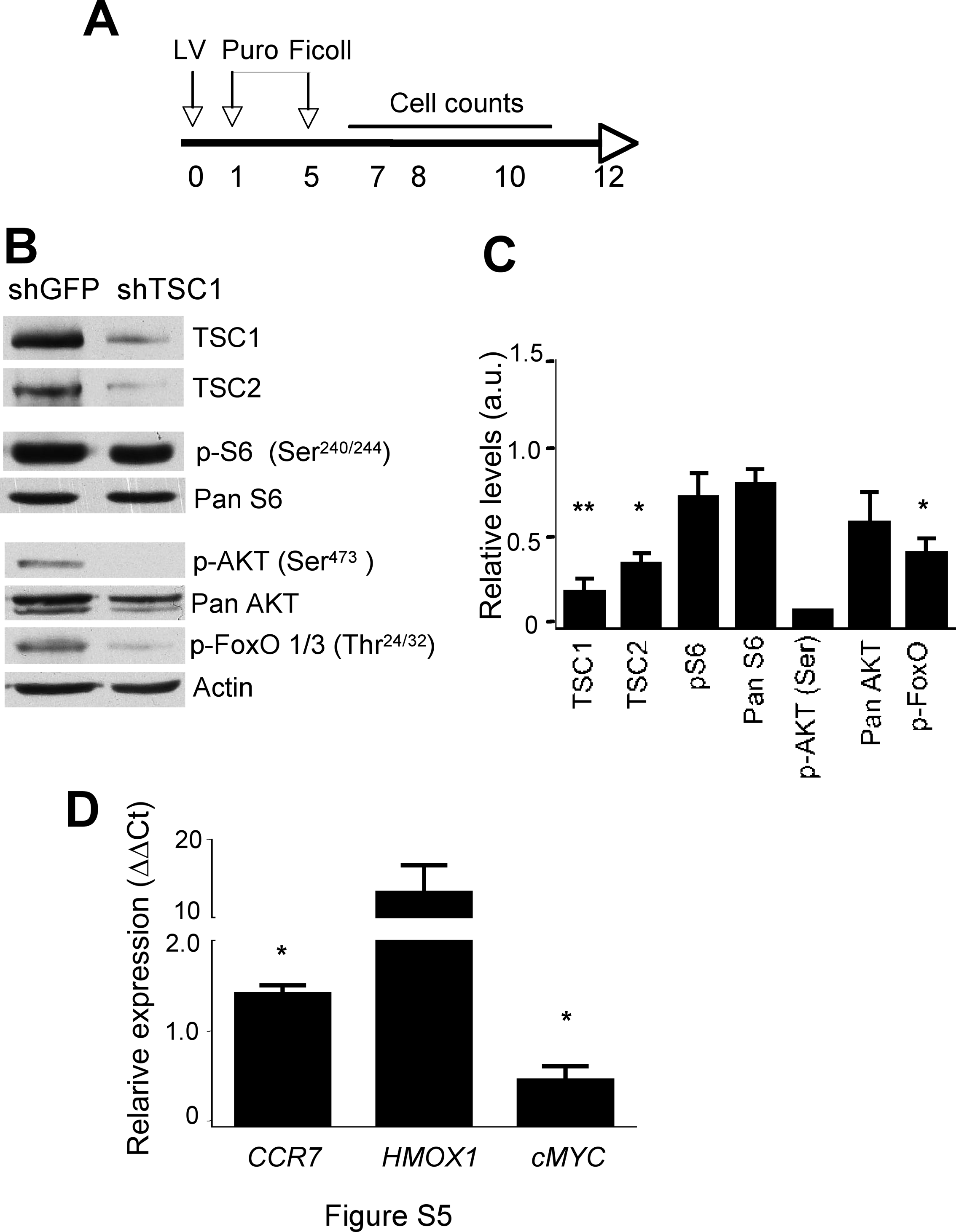

Supplement: Figure S5 — shRNA-assisted TSC1 knock down hinders mTORC2 dependent regulation of FoxO1/3 in transformed Jurkat T cells. Jurkat T-leukemia cells were transduced by lentiviral-encoding TSC1 shRNA or scrambled control GFP-tagged shRNA. Cells were selected for 4 days in Puromycin. By then, no viable cells could be recovered from untransduced Puromycin-treated cells. Viable transduced cells separated on a Ficoll gradient, counted and re-plated in fresh complete medium. A) Schematic representation of the cell transduction/selection. B–C) Scrambled shRNA (GFP) and TSC1 shRNA-infected cells (shTSC1) were analyzed for WB. In C, densitometry analyses of three experiments are shown. Data were first normalized by Actin, and then expressed relatively to GFP control cells. D) Expression of the indicated FoxO targets was measured by Real time PCR. Data were normalized to the expression of the housekeeping gene (GAPDH) and expressed relatively to control cells (GFP) by the ΔΔCt method. Statistical significance was determined by a paired, two-tailed Student t-Test. (TIF) [file pone.0091952.s005.tif]

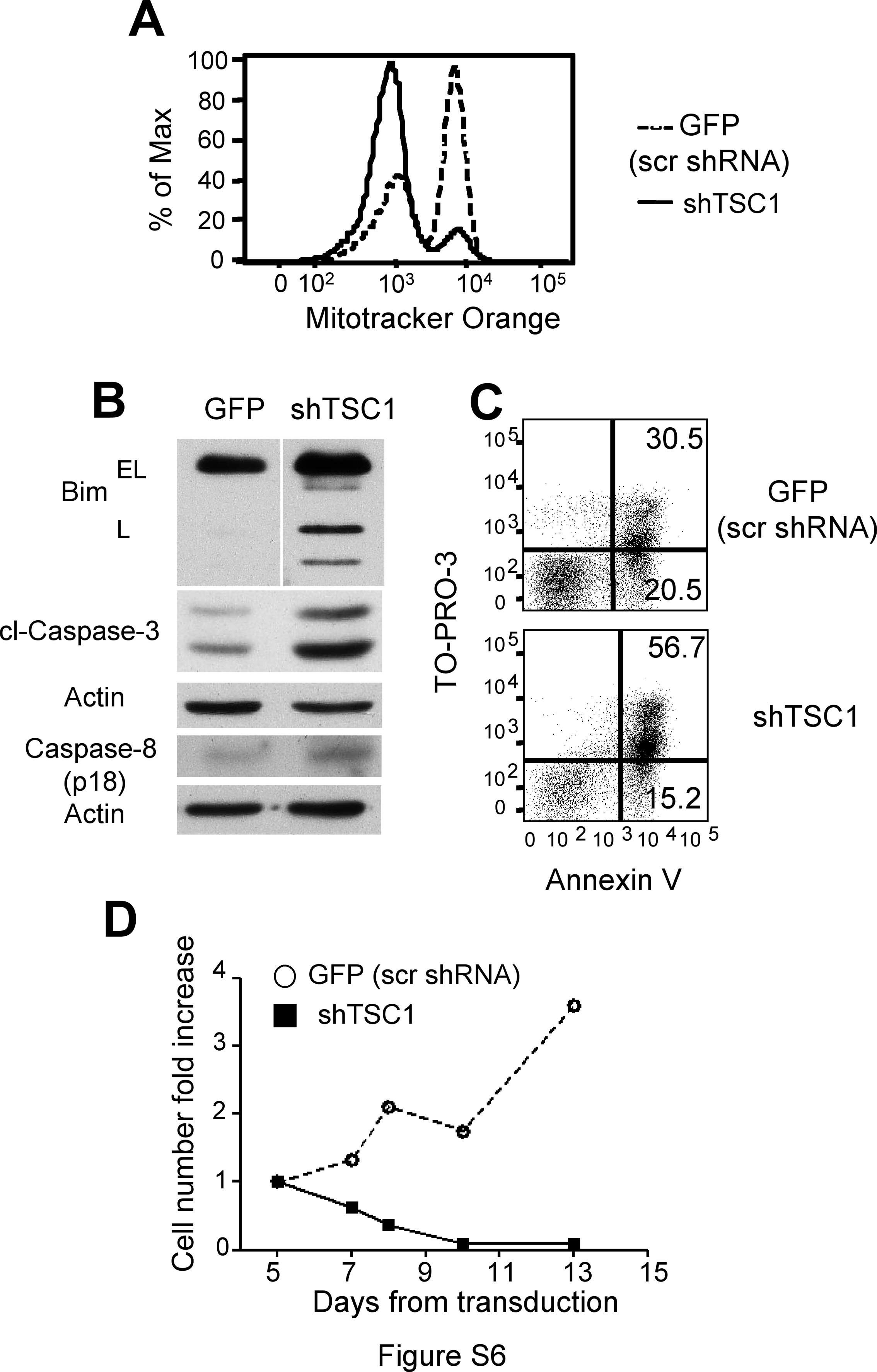

Supplement: Figure S6 — shRNA-assisted TSC1 knock down hinders survival of transformed Jurkat T cells. GFP and shTSC1 cells were obtained as indicated in Figure S5A. Cells were harvested at day 8 (three days after Ficoll) and stained with the Mitotracker Orange dye (A), or with Annexin V (C) and analyzed by flow cytomerty, or lysed and analyzed by Western blot (B). Histogram overlays (A) and dot plots (C) are representative of 4 and 3 independent experiments, respectively. In B, WB images representative of 2–3 independent determinations are shown. D) Cell viability was determined over time by Trypan blue-assisted counts. Data are depicted as fold changes over input and are representative of one of three independent similar determinations. (TIF) [file pone.0091952.s006.tif]
