# Supplementary material for: Monoallelic Germline TSC1 Mutations Are Permissive for T Lymphocyte Development and Homeostasis in Tuberous Sclerosis Complex Individuals
Source: PLoS One. 2014 Mar 14;9(3):e91952. doi: 10.1371/journal.pone.0091952 (PMC3954840; doi:10.1371/journal.pone.0091952)
Supplement: Table S1 — Clinical manifestations of TSC in the analyzed sample group. (DOC) [file pone.0091952.s007.doc]

**TableS1. Clinical manifestations of TSC in the analyzed sample group.**

|  | Age/sex | Hypomel macules | Facial  angiofib | Periungual  fibroma | Shagree  patches | Angio  miolip | Skin tags | Lung/  kidney | Tubers | Mental  Ret/epil |
| --- | --- | --- | --- | --- | --- | --- | --- | --- | --- | --- |
| Pt1 | 58y/male | + | + | + | + | - | + | -/- | - | - |
| Pt2 | 30y/male | + | + | + | + | - | - | -/- | + | mild/+ |
| Pt3 | 52y/male | + | + | + | - | + | + | +/- | + | - |
| Pt4 | 49y/male | - | + | + | - | + | + | +/+ | + | - |

Clinical manifestations at the time of diagnosis, and over the course of follow up included the scoring of hypomelanotic macules, facial angiofibomas, periungual fibroma, shagree, angiomiolipoma, skin tags, lung and kidney nodules, brain tubers, and signs of mental retardation or epilepsy.
