# Supplementary material for: Correction: Monoallelic Germline TSC1 Mutations Are Permissive for T Lymphocyte Development and Homeostasis in Tuberous Sclerosis Complex Individuals
Source: PLoS One. 2019 Jun 7;14(6):e0218354. doi: 10.1371/journal.pone.0218354 (PMC6555530; doi:10.1371/journal.pone.0218354)

PONE-S-13-55189

Figure 2A

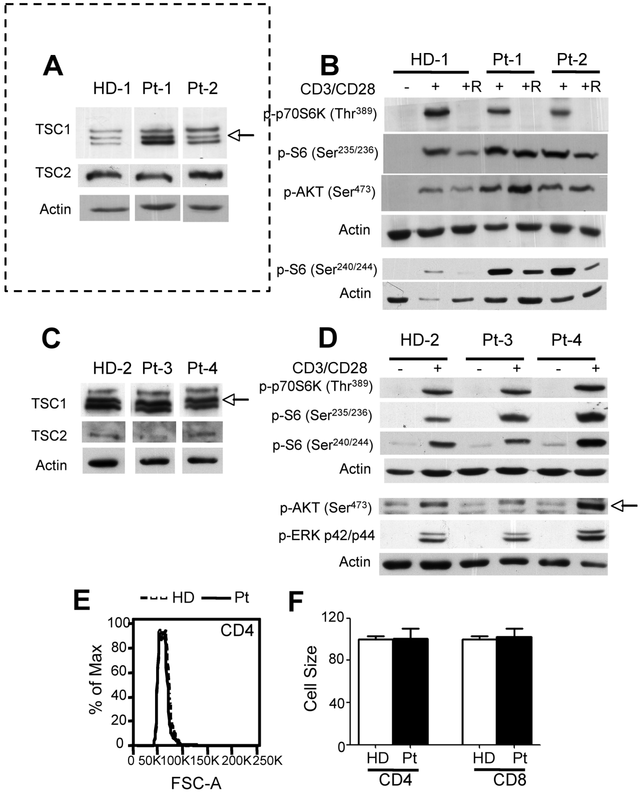

Figure 2

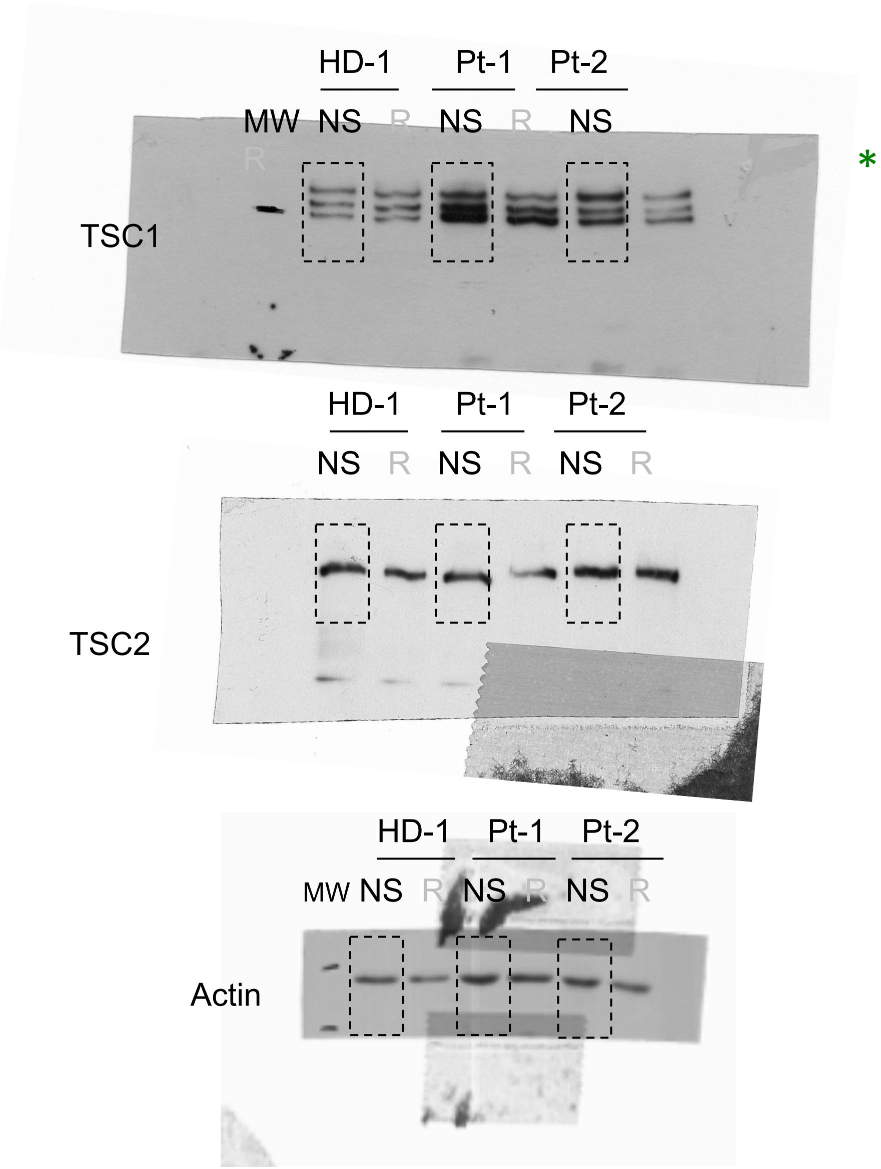

** -NB new scan*

PONE-S-13-55189

Figure 2B

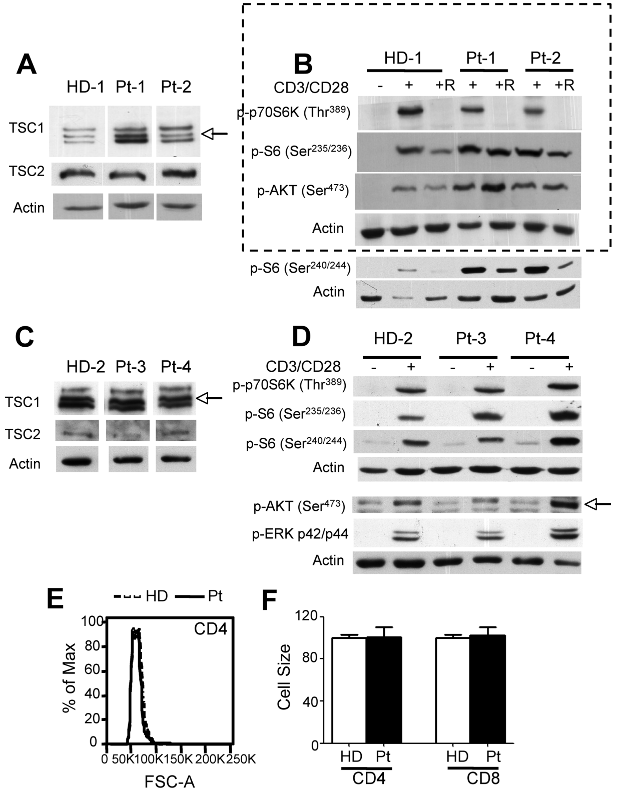

Figure 2

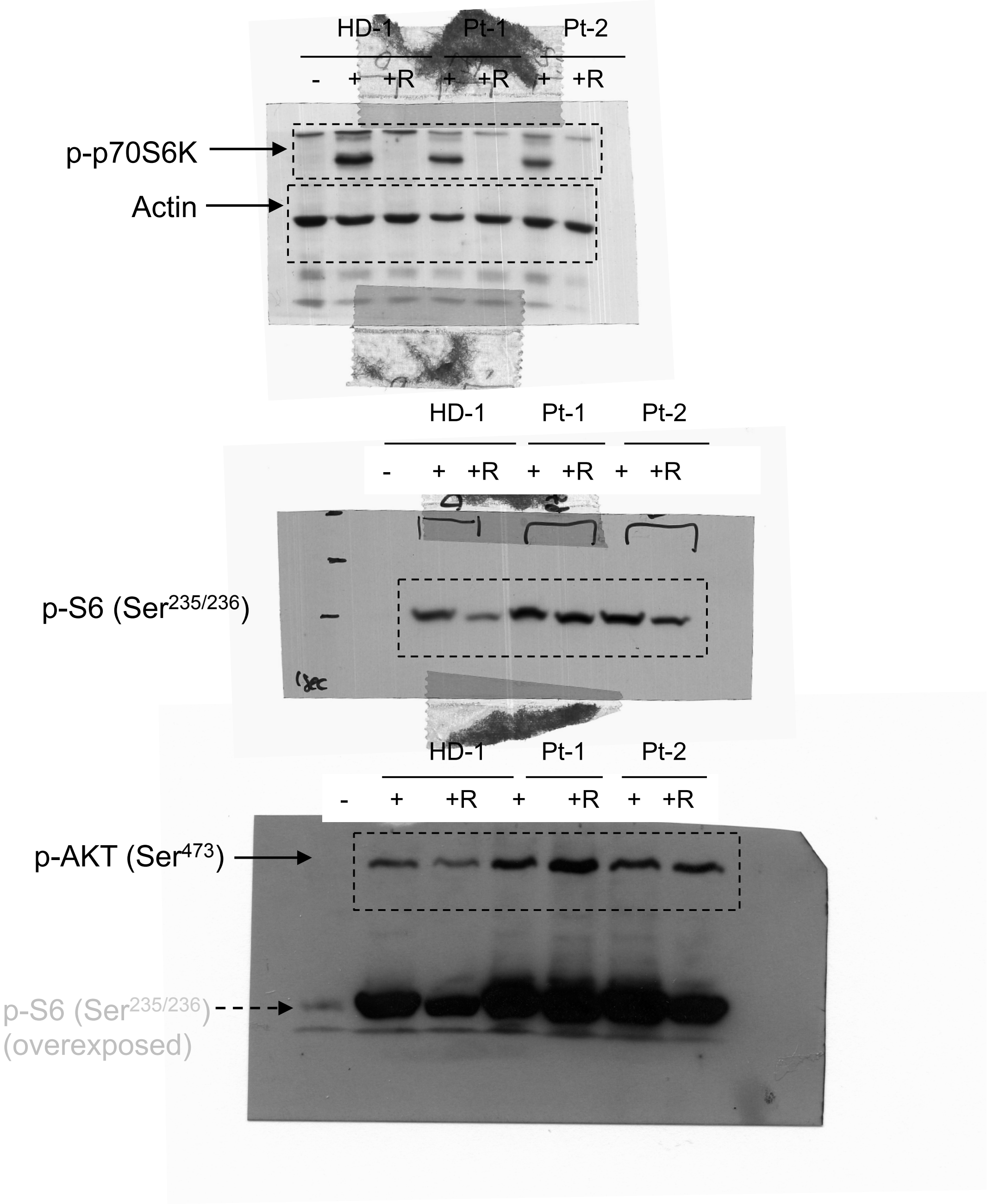

PONE-S-13-55189
Figure 2B (continue)

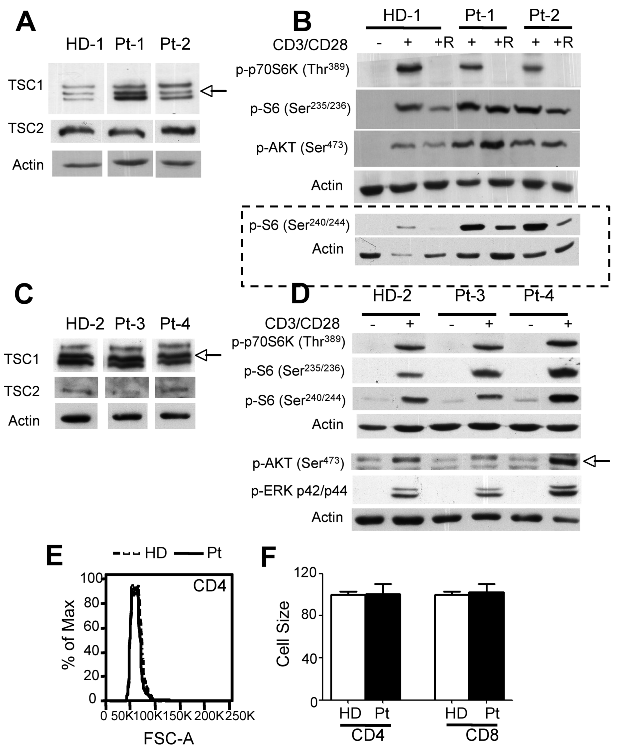

Figure 2

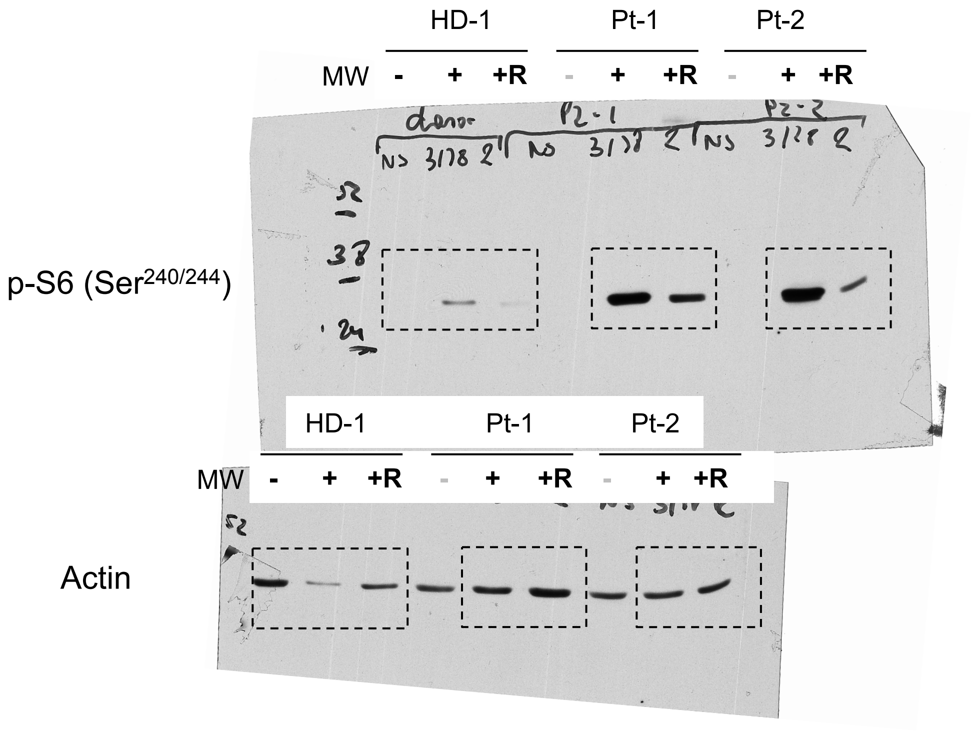

PONE-S-13-55189
Figure 2C

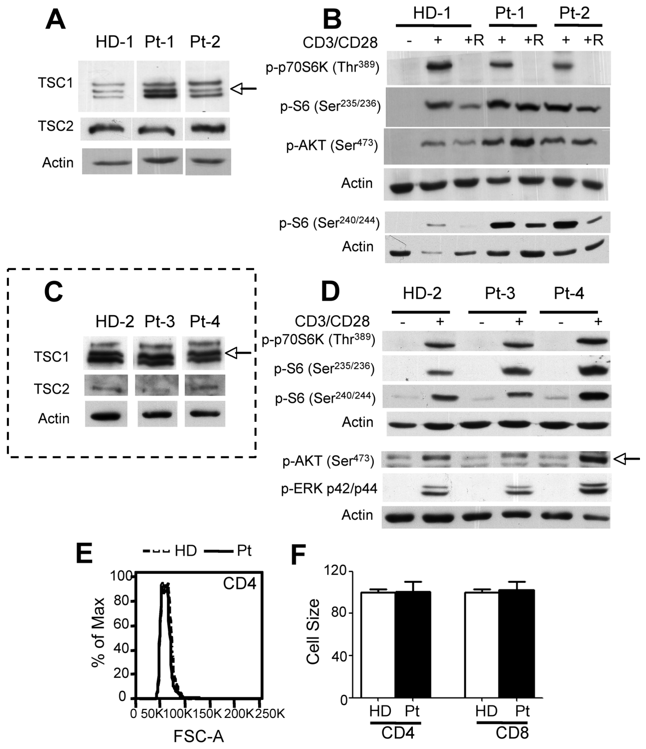

Figure 2

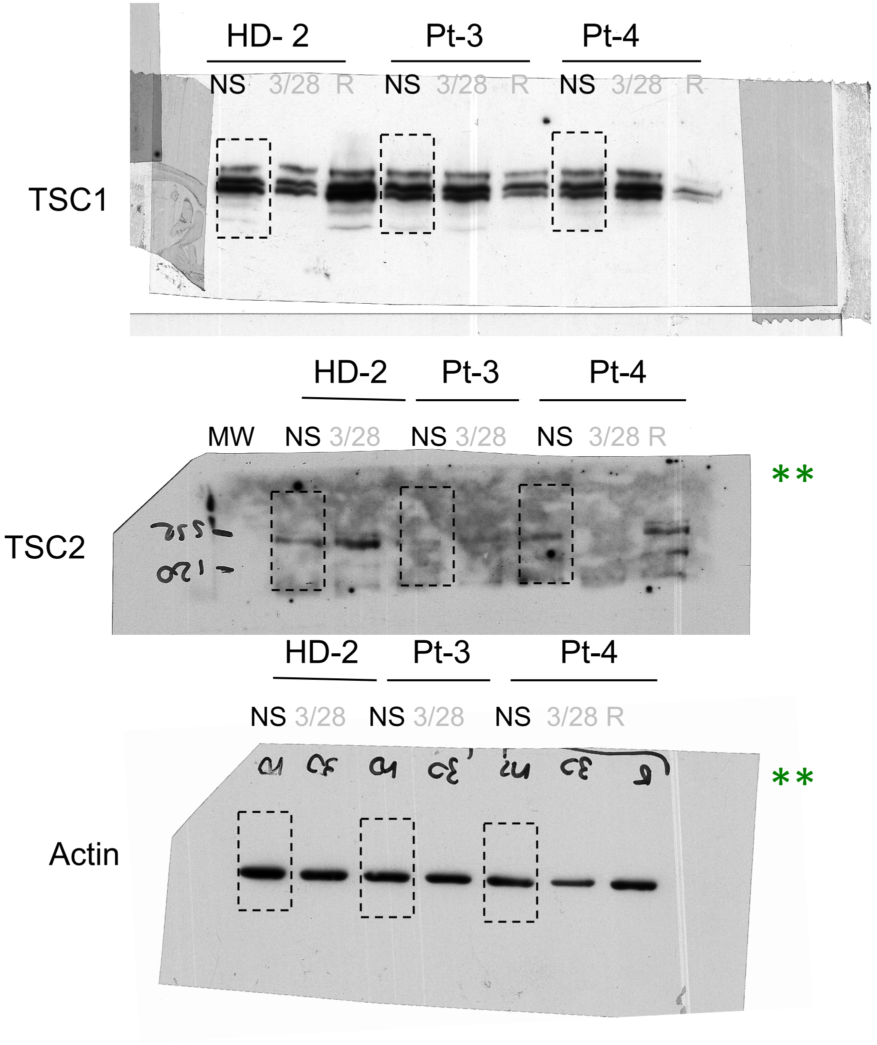

*** - NB original scans were flipped horizontally to properly reflect sample order*

PONE-S-13-55189
Figure 2D (revised)

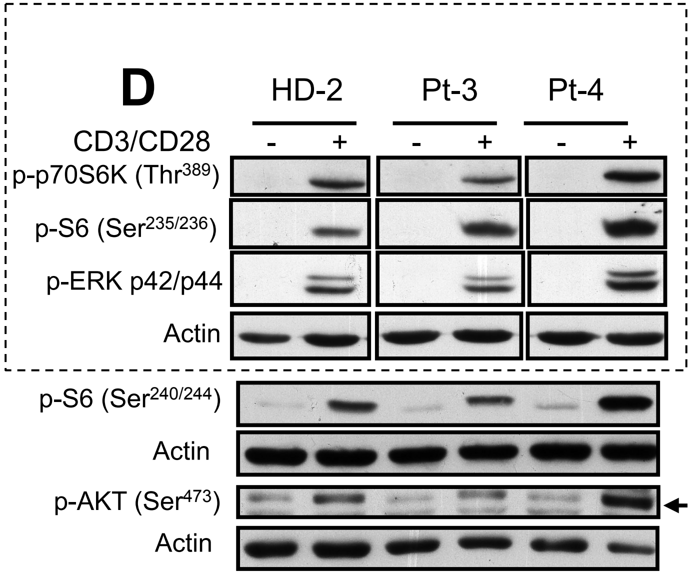

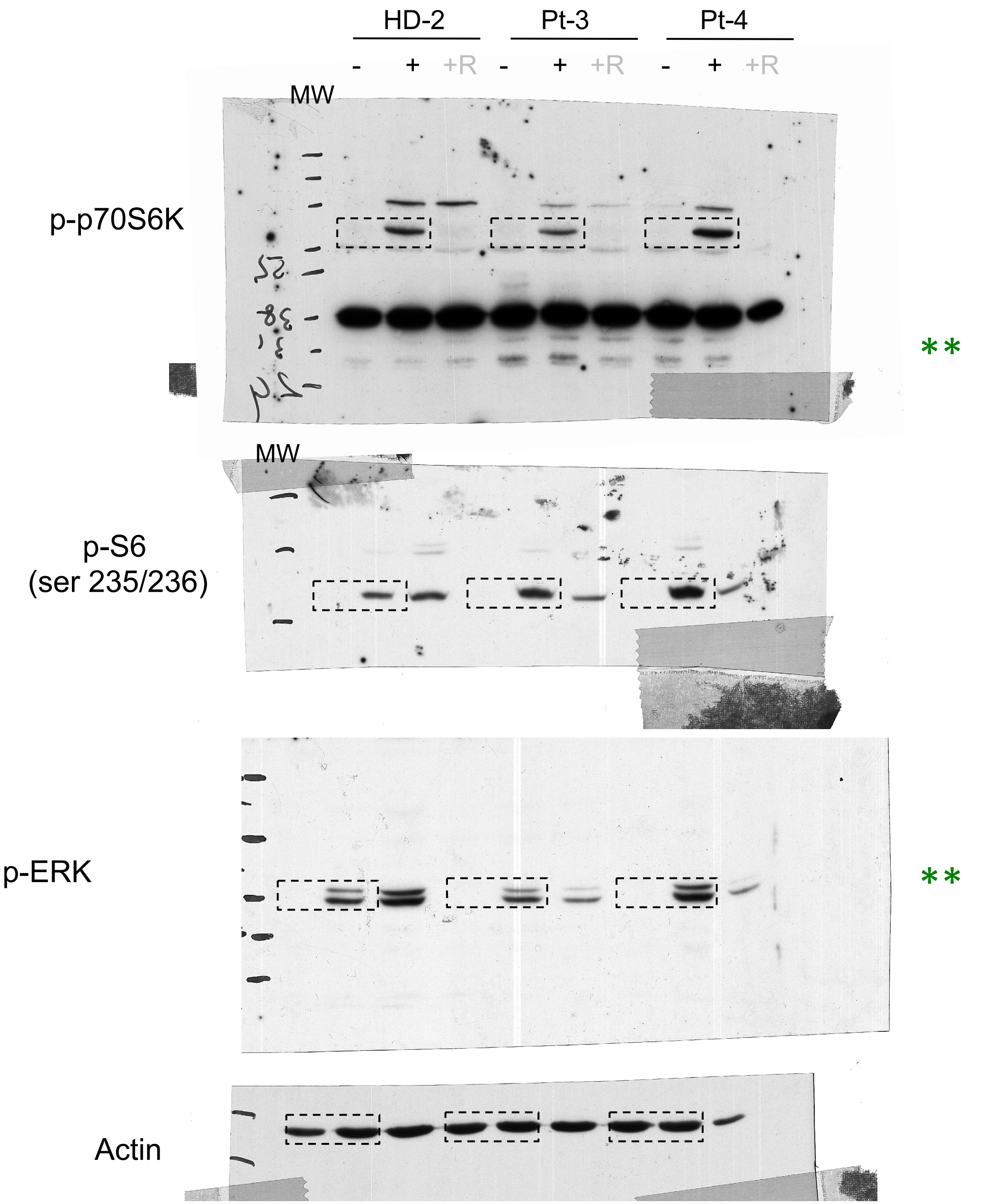

*** - NB original scans were flipped horizontally to properly reflect sample order*

PONE-S-13-55189 Figure 2D (revised, continue)

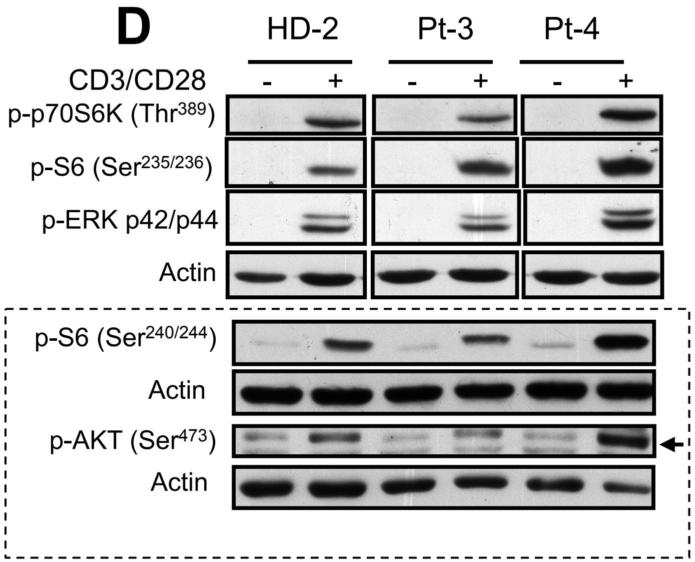

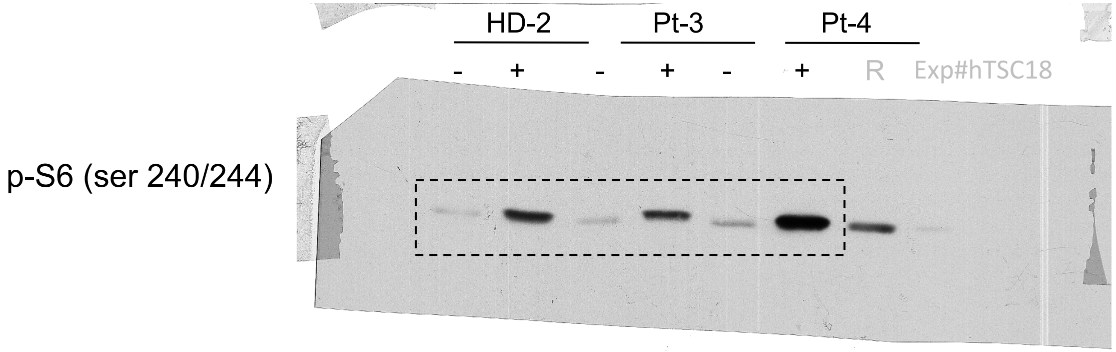

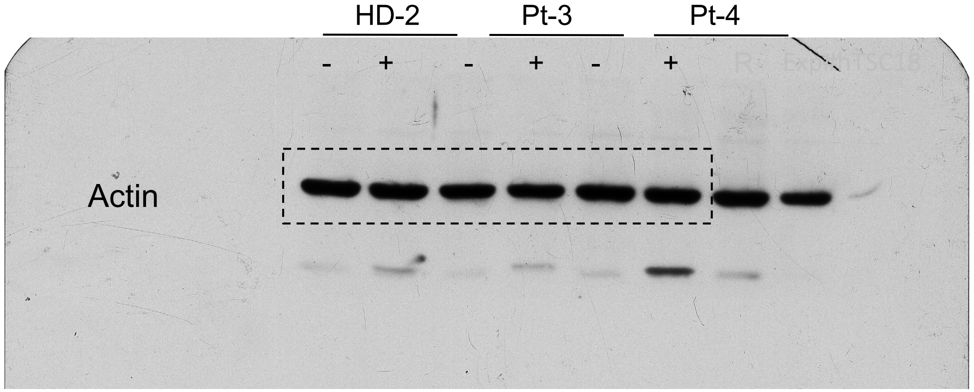

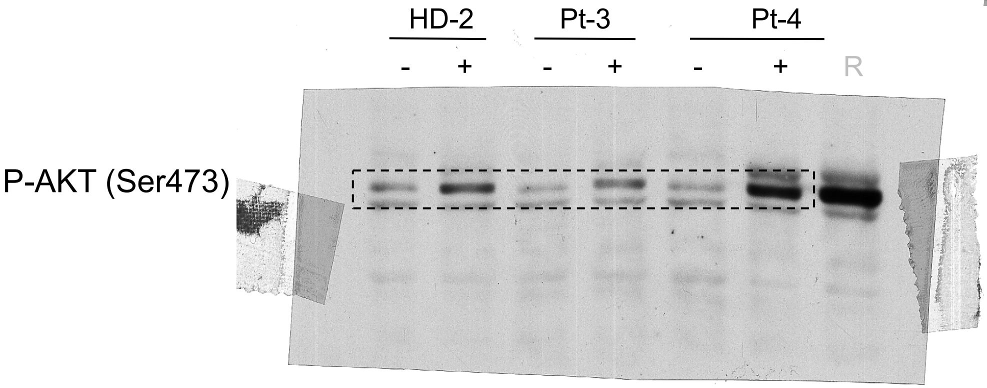

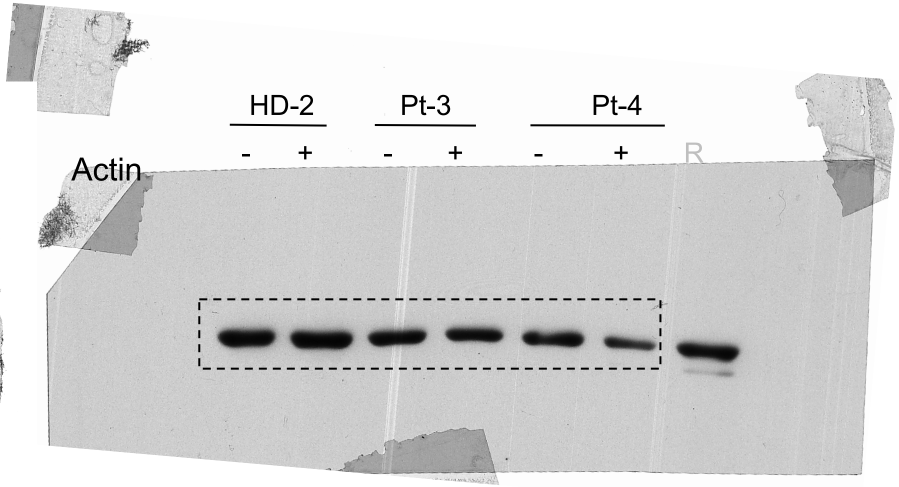

Supplement: S1 File — The raw data used to assemble panel A-D of Fig 2 are depicted. Dotted lines reflect the portion of the images that were used to assemble the final panels. No image adjustment was used. (PDF) [file pone.0218354.s001.pdf]
