# Supplementary material for: Correction: Monoallelic Germline TSC1 Mutations Are Permissive for T Lymphocyte Development and Homeostasis in Tuberous Sclerosis Complex Individuals
Source: PLoS One. 2019 Jun 7;14(6):e0218354. doi: 10.1371/journal.pone.0218354 (PMC6555530; doi:10.1371/journal.pone.0218354)
Supplement: S3 File — The raw data used to assemble panels B and C of Fig 4 and pane B of Fig 5 are depicted. Dotted lines reflect the portion of the images that were used to assemble the final panels. No image adjustment was used. (PDF) [file pone.0218354.s003.pdf]

PONE-S-13-55189
Figure 4B

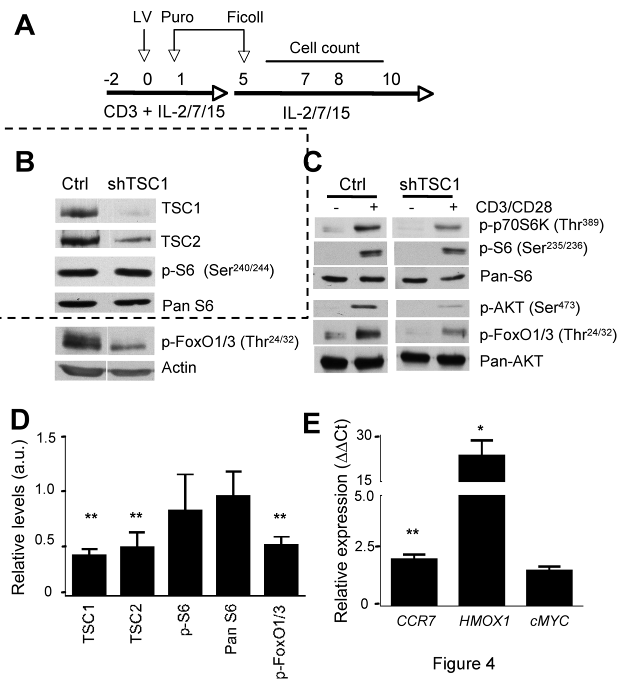

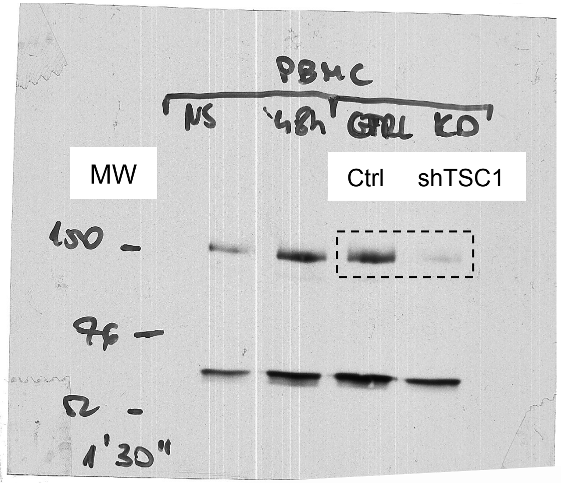

TSC1

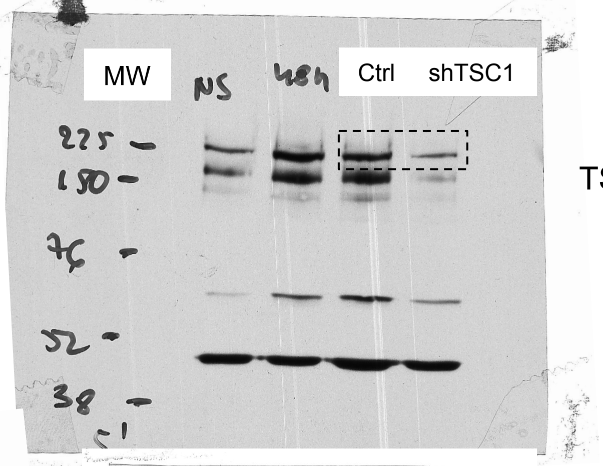

TSC2

Pan S6

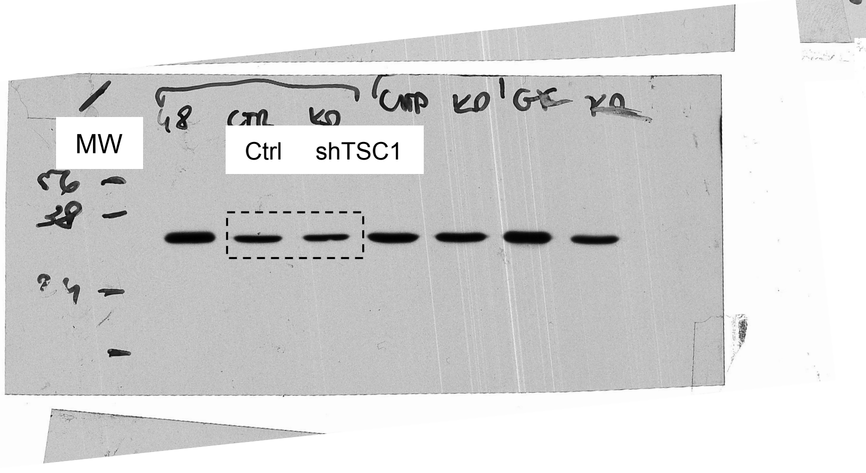

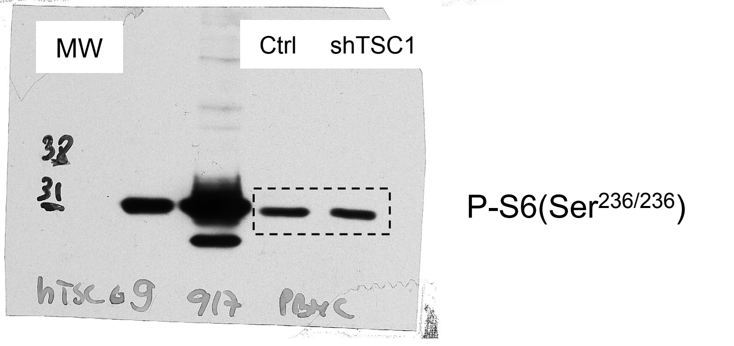

P-S6(Ser$^{236/236}$)

PONE-S-13-55189
Figure 4B (continue)

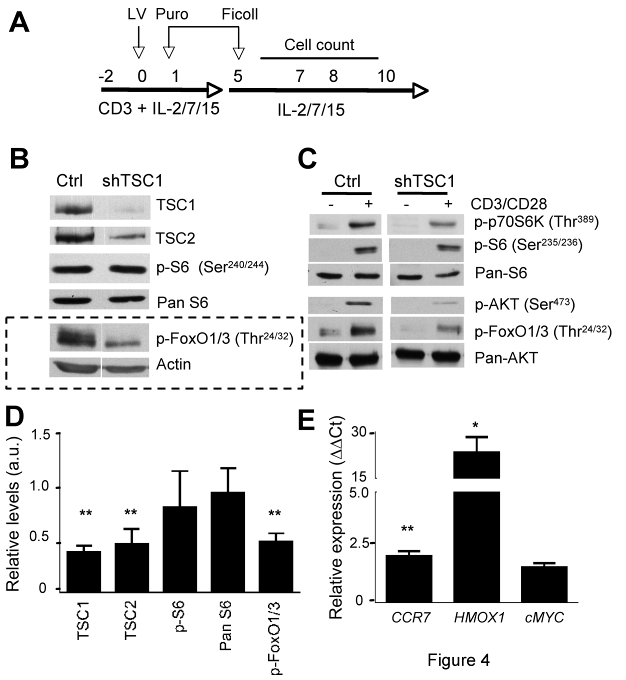

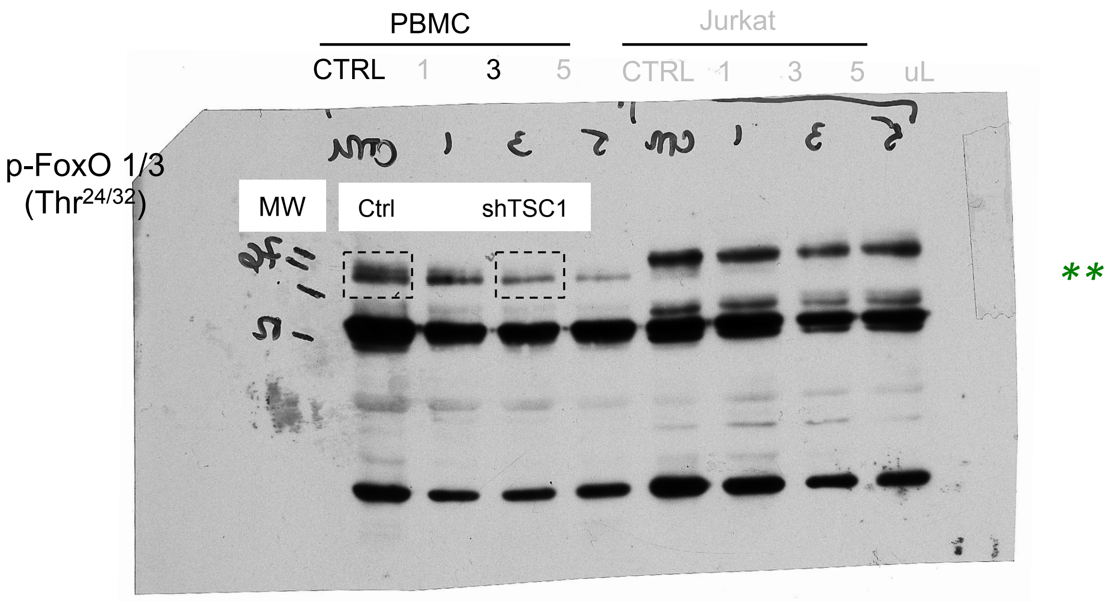

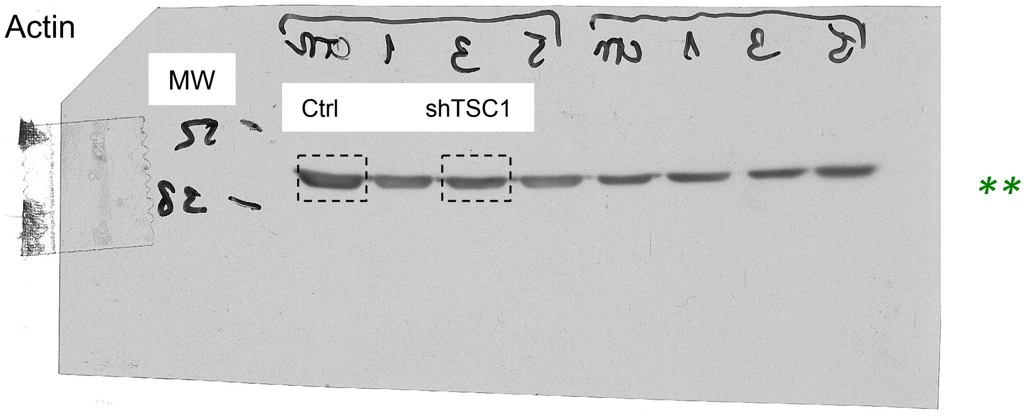

*** - NB original scans were flipped horizontally to properly reflect sample order*

PONE-S-13-55189
Figure 4C

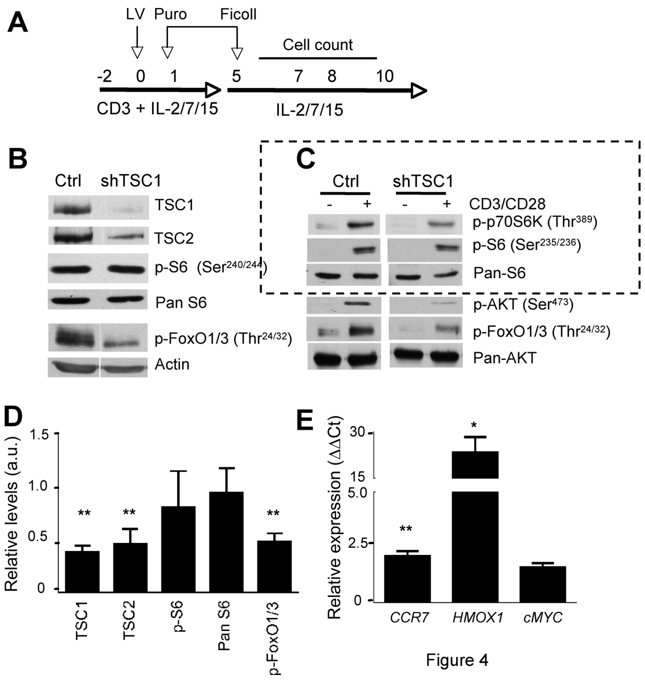

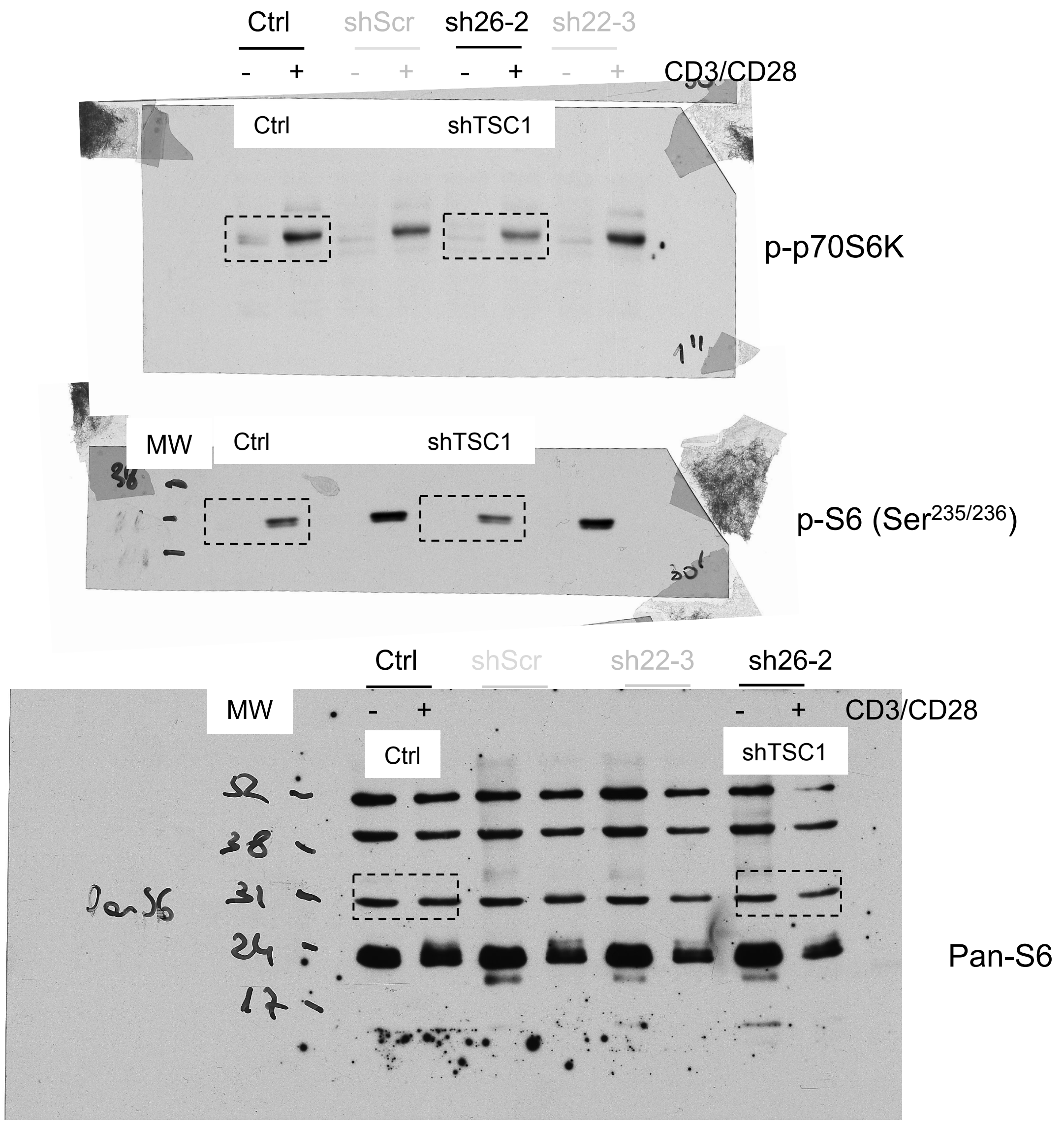

PONE-S-13-55189
Figure 4C (continue)

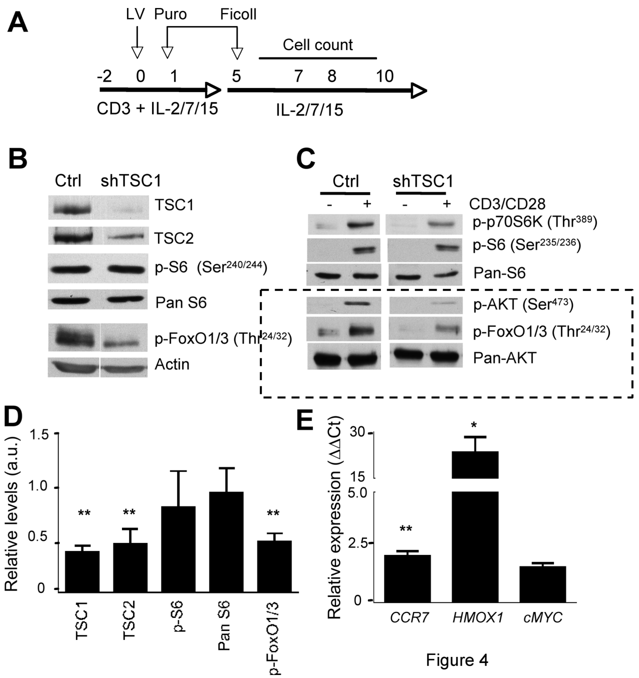

Figure 4

p-AKT (Ser473)

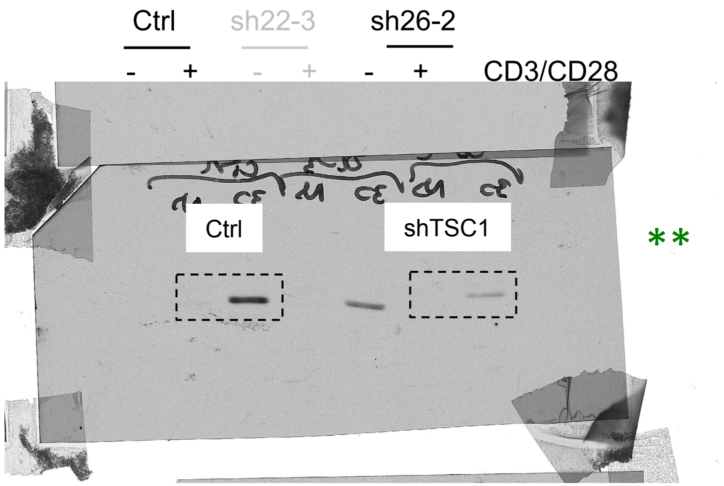

**

p-FoxO 1/3 (Thr24/32)

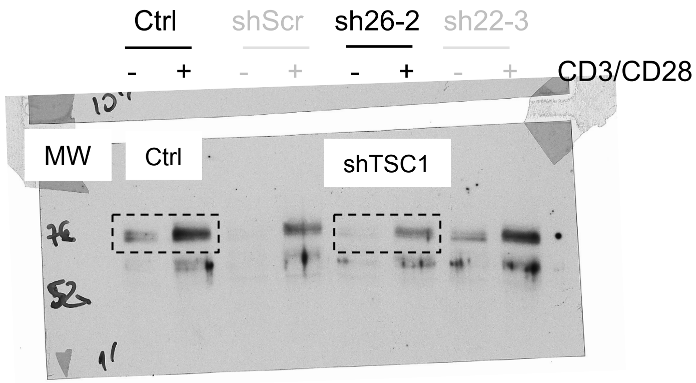

Pan-AKT

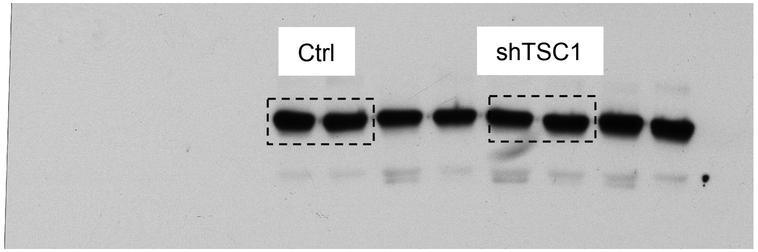

*** - NB original scans were flipped horizontally to properly reflect sample order*

PONE-S-13-55189
Figure 5B

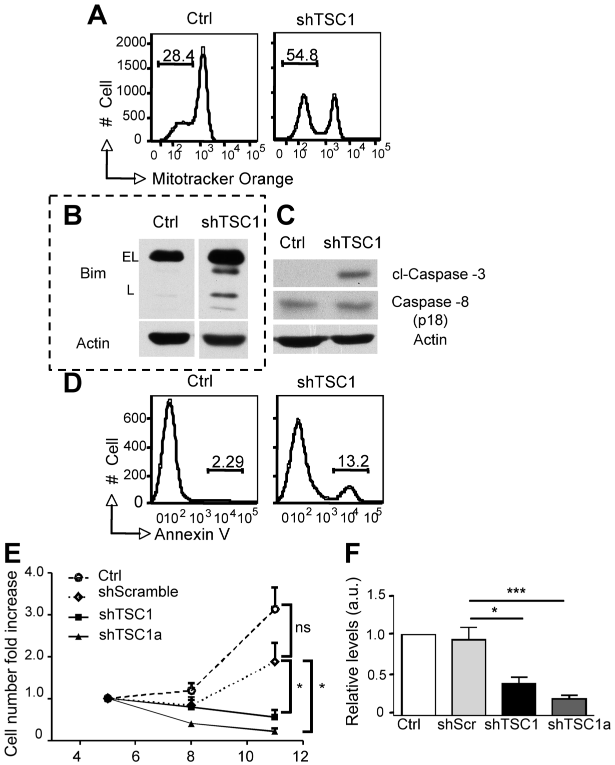

Figure 5

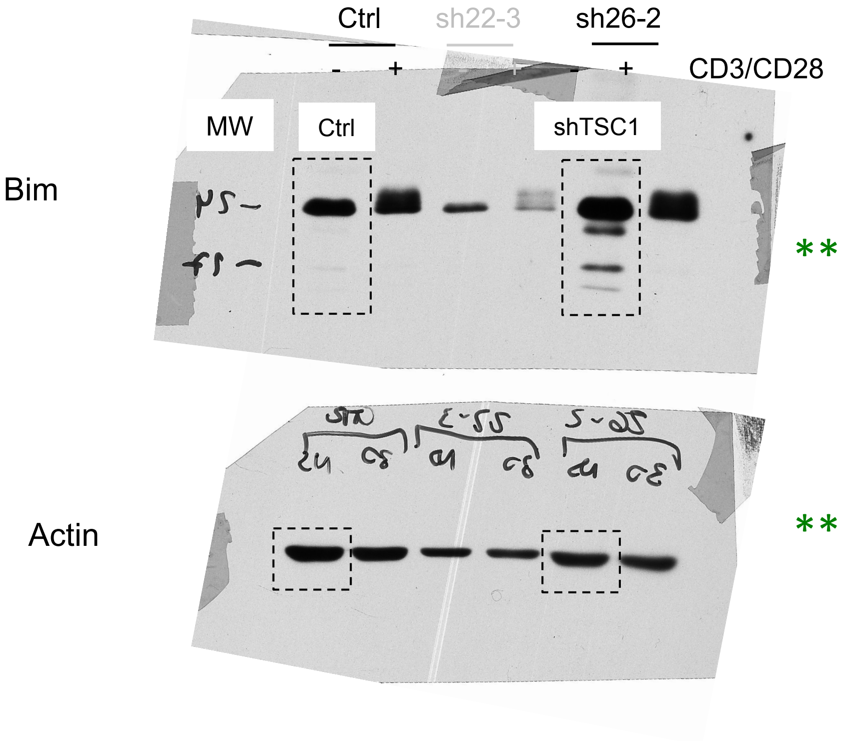

*** - NB original scans were flipped horizontally to properly reflect sample order*
